# Halogen bonding relay and mobile anion transporters with kinetically controlled chloride selectivity†

**DOI:** 10.1039/d3sc01170d

**Published:** 2023-04-04

**Authors:** Toby G. Johnson, Andrew Docker, Amir Sadeghi-Kelishadi, Matthew J. Langton

**Affiliations:** a Chemistry Research Laboratory, Department of Chemistry, University of Oxford 12 Mansfield Road Oxford OX1 3TA UK matthew.langton@chem.ox.ac.uk

## Abstract

Selective transmembrane transport of chloride over competing proton or hydroxide transport is key for the therapeutic application of anionophores, but remains a significant challenge. Current approaches rely on enhancing chloride anion encapsulation within synthetic anionophores. Here we report the first example of a halogen bonding ion relay in which transport is facilitated by the exchange of ions between lipid-anchored receptors on opposite sides of the membrane. The system exhibits non-protonophoric chloride selectivity, uniquely arising from the lower kinetic barrier to chloride exchange between transporters within the membrane, compared to hydroxide, with selectivity maintained across membranes with different hydrophobic thicknesses. In contrast, we demonstrate that for a range of mobile carriers with known high chloride over hydroxide/proton selectivity, the discrimination is strongly dependent on membrane thickness. These results demonstrate that the selectivity of non-protonophoric mobile carriers does not arise from ion binding discrimination at the interface, but rather through a kinetic bias in transport rates, arising from differing membrane translocation rates of the anion–transporter complexes.

## Abstract 2

A halogen bonding anion relay with Cl^−^ > OH^−^ selectivity is reported. Selectivity is maintained across lipid membranes of different thicknesses, whereas for selective mobile carriers the discrimination is strongly dependent on membrane thickness.

## Graphical abstract

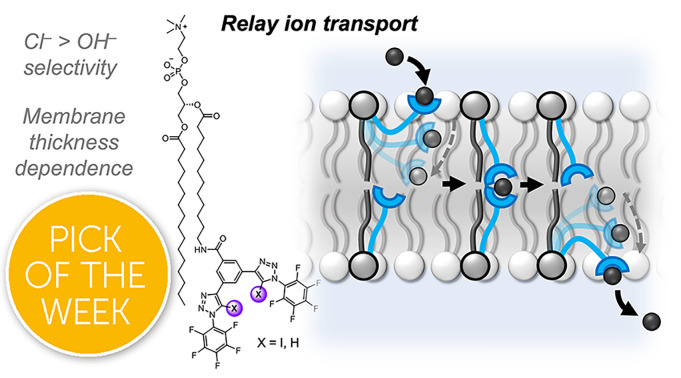

## Introduction

Transmembrane ion transport in nature is mediated by protein channels and pumps which span the membrane and achieve remarkable ion transport selectivity.^1,2^ Diseases associated with compromised ion channel function, including cystic fibrosis and Best's disease, have motivated the development of synthetic ion transporters as potential therapeutics, particularly for anions.^3–8^ Significant efforts have focused on discrete molecular anion carriers (anionophores) with the overwhelming majority exploiting hydrogen bonding (HB) donor arrays for anion complexation.^9–13^ Achieving high non-protonophoric chloride anion selectivity (Cl^−^ > OH^−^/H^+^) in these systems is key for downstream therapeutic applications, to avoid disruption of cellular pH gradients.^14^ In the context of anion supramolecular chemistry, sigma-hole interactions^15,16^ such as halogen bonding (XB) and chalcogen bonding (ChB) have come to the fore as powerful alternative non-covalent interactions.^17–21^ Indeed, recent reports have demonstrated that XB and ChB integration into mobile carrier design is accompanied with numerous advantages,^22–27^ including redox–controllable activity^28–30^ and chloride over hydroxide selectivity.^31,32^

**Fig. 1:**
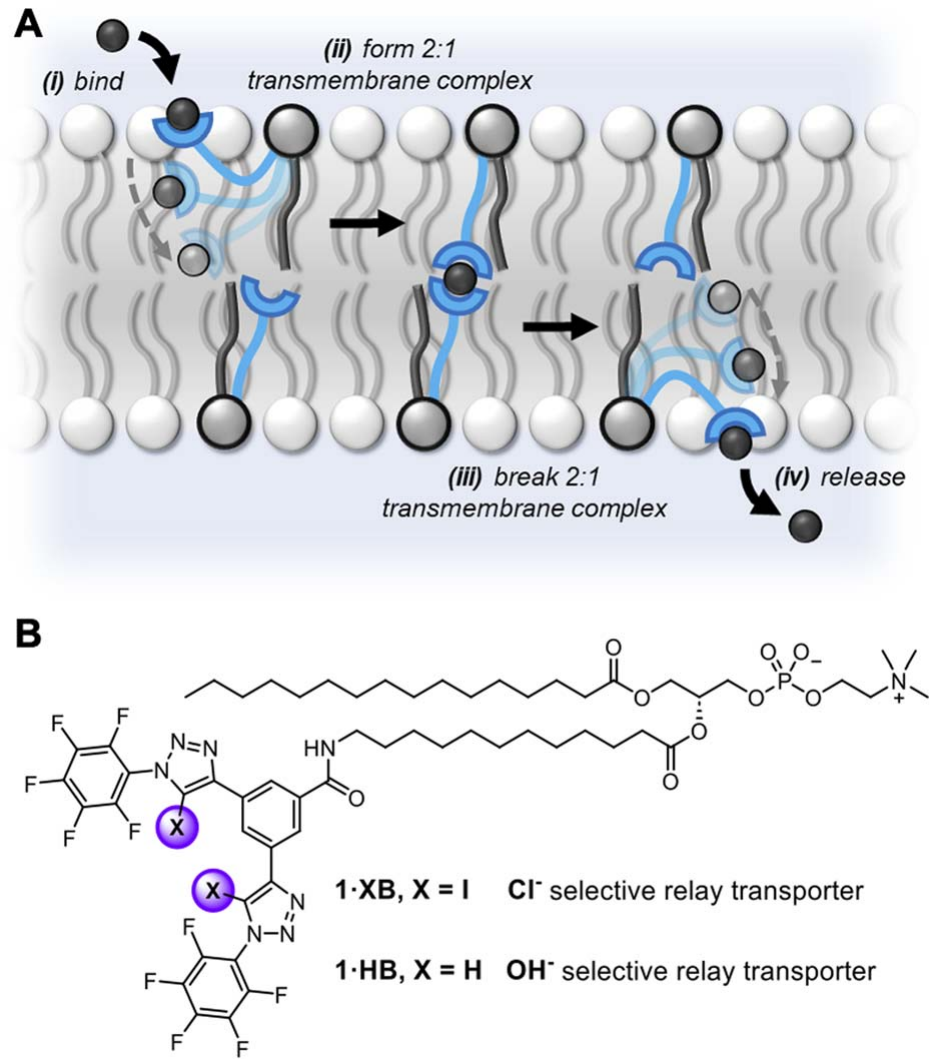
(A) Schematic representation of the relay transport mechanism. (B) Structure of the lipid anchored XB and HB relay transporters 1·XB and 1·HB, respectively.

While nature employs either mobile carriers or channels to mediate transport across cellular membranes, new mechanisms of ion transport based on *membrane-anchored carriers* have demonstrated considerable promise.^33^ These abiotic anchored carriers can be subdivided into two classes: *unimolecular*^34–39^ and *relay transporters* (Fig. 1A).^40–42^ In the former, an individual ion carrier is tethered to a membrane anchoring unit with a sufficiently long linker such that it is capable of reaching across the bilayer and mediating ion transport *via* a carrier-like mechanism.^43^ Examples include molecular ion fishers,^38,39^ swing transporters,^36,37^ and rotaxane-based shuttles.^34,35^ In contrast, relay transport, as originally demonstrated by Smith,^41^ requires two anchored ion receptors in opposite leaflets of the bilayer to facilitate the exchange of the ion across the membrane interior. More recently we have developed a relay transport system in which the activity is regulated by photo-isomerisation of the transporters within the membrane.^42^ Anchoring an ion carrier as part of a phospholipid is advantageous because it provides an amphiphilic transport system which should enable enhanced formulation and delivery in future therapeutic applications, unlike typical lipophilic mobile ion carriers. Notwithstanding these reports, examples of relay transporters are extremely rare and arguably constitute the most underdeveloped synthetic transporter system. Motivated by the advantages of relay-based transport and sigma-hole mediated anion recognition, particularly for achieving chloride-selective transport, we sought to combine these aspects in the design of an XB membrane-anchored relay transporter.

Herein, we report the first example of a relay ion transporter utilising XB anion binding interactions. We show that this system is two orders of magnitude more active than the previous highest performing relay transporter, with significant selectivity for Cl^−^ > OH^−^. We explore the origin of this selectivity in comparison with analogous chloride-selective non-protonophoric mobile carriers and demonstrate that the anion selectivity in these carriers arises from kinetic factors. The preferential rate of translocation of the chloride-carrier complex across the membrane compared to that of the hydroxide-carrier complex contributes to the selectivity, which is strongly dependent on the thickness of the lipid bilayer membrane.

## Results and discussion

### Design and synthesis

The targeted XB relay transport system, 1·XB, features a phosphatidylcholine lipid scaffold that serves to anchor the system within the lipid membrane. The polar zwitterionic headgroup inhibits membrane translocation and thus confines the transporter to one membrane leaflet.^41,42^ The perfluoroaryl functionalised bis-iodotriazole motif acts as the anion binding domain,^32,44^ wherein the integration of perfluorinated moieties enhances both XB donor potency and receptor lipophilicity.^45,46^ A HB bis-prototriazole analogue, 1·HB, was also prepared for means of comparison. Full synthetic details for the preparation of the transporters and characterisation are included in the ESI (Fig. S1–S21†).

### Relay mediated ion transport

The transport activity of the XB and HB relay transporters 1·XB and 1·HB, respectively, was established using ion transport assays in large unilamellar vesicles (LUVs). The pH-responsive fluorophore 8-hydroxypyrene-1,3,6-trisulfonate (HPTS) was encapsulated within 200 nm 1-palmitoyl-2-oleoyl-*sn*-glycero-3-phosphocholine vesicles (POPC LUVs) in NaCl solution, buffered to pH 7.0 with 4-(2-hydroxyethyl)piperazine-1-ethanesulfonic acid (HEPES). Pre-incorporating the relay transporter during LUV preparation generates an equal distribution of the transporter in both leaflets of the membrane. Addition of an external base pulse (NaOH, 5 mM) generates a pH gradient, which is dissipated by transporter-mediated Cl^−^/OH^−^ antiport (or the functionally equivalent H^+^/Cl^−^ symport). The process was monitored by recording the change in HPTS emission, *I*_rel_ (*λ*_em_ = 510 nm), with time following excitation at *λ*_ex_ = 405/460 nm. The addition of detergent (Triton X-100) facilitated calibration of the emission intensity.

**Fig. 2:**
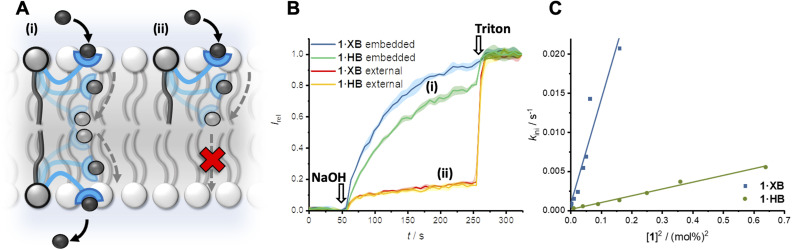
(A) Relay anion transport. (B) Change in ratiometric emission, *I*_rel_ (*λ*_em_ = 510 nm; *λ*_ex1_ = 405 nm, *λ*_ex2_ = 460 nm), upon addition of a NaOH base pulse (5 mM) to POPC LUVs (31 μM) containing 1 mM HPTS, 100 mM internal and external NaCl, buffered with 10 mM HEPES at pH 7.0. (i) Data for relay transporter pre-incorporated during LUV preparation (0.25 mol% 1·XB with respect to lipid and 1 mol% 1·HB) and (ii) following external addition of 1 mol% relay transporter in DMSO (>95% membrane incorporation efficiency). (C) Concentration dependence of relay activity with pre-incorporated 1·XB and 1·HB, and linear relationship for the initial rate, *k*_ini_ with respect to [1]^2^.

The XB and HB relays proved to be effective anion transporters when incorporated into both leaflets of the membrane (Fig. 2A and B). In contrast, when the relays were positioned in only the outer leaflet of the membrane – by addition of the relay transporter in DMSO to pre-formed LUVs – no detectable ion transport was observed. Membrane uptake at >95% efficiency was confirmed by UV-vis experiments (Fig. S22†). This confirms the requirement for relay transporters to be present in both leaflets of the membrane to complete the transmembrane transport process, and that the lipid anchor prevents membrane translocation of the anchored transporter from the outer to the inner leaflet.

A non-linear dependence of the observed initial anion transport rate, *k*_ini_, on the concentration of pre-incorporated relay transporter was observed for both 1·XB and 1·HB, which is characteristic of multiple molecules implicated in the rate-determining step of ion transport (Fig. 2C). A relay mechanism requires two transporter molecules, with one in each leaflet of the membrane. The observed linear relationship of *k*_ini_*versus* [1]^2^ is consistent with this mechanism, and implies the exchange step between transporters in opposite leaflets is rate-limiting. The same linear relationship was observed for both 1·XB and 1·HB indicating that both facilitate relay transport with the same bimolecular rate-determining step. Hill analysis of the dose response curves enabled quantification of the transport activity of each transporter through an effective concentration value (EC_50_) required to reach 50% activity, of 0.18 mol% and 0.59 mol% for 1·XB and 1·HB in POPC LUVs, respectively. The corresponding Hill coefficients of 2.8 and 4.8 are consistent with multiple relay transporters in the rate-limiting transport process, but it should be noted that the absolute values are very sensitive to conditions and minor structural changes and provide minimal information about stoichiometry compared to kinetics analysis.^47^ The XB relay 1·XB outperforms the HB analogue 1·HB by a factor of three, consistent with previous reports of XB enhanced anion affinity relative to HB prototriazole equivalents,^32^ and to the best of our knowledge constitutes the most active relay transporter reported to date.

The mechanism of pH dissipation is likely dominated by Cl^−^/OH^−^ antiport, with the functionally equivalent Cl^−^/H^+^ symport improbable given the low basicity of the triazole anionophores (p*K*_a_*H* ∼0–1),^48^ in agreement with observations from previous studies on XB-mediated anion transport.^26,32^ Transport was not detected when chloride was replaced with gluconate, a larger hydrophilic anion, which is consistent with 1·XB being incapable of either cation transport (*via* H^+^/Na^+^ antiport) or overcoming the significant dehydration penalty required for a OH^−^/gluconate antiport process (Fig. S26†). Anion transport activity of 1·XB in the lipid gel phase of dipalmitoylphosphatidylcholine (DPPC) LUVs at 25 °C was arrested, and restored when heated to 45 °C, above the gel–liquid phase transition temperature (*T*_m_ = 41 °C, Fig. S27†). This behaviour is consistent with the proposed relay transport mechanism, in which mobility of the relay “arms” through the lipid bilayer is required, and hence transport capability is dramatically reduced in the gel phase. Inhibition of relay anion transport by 1·XB in anionic phosphoglycerol lipids (Fig. S28†) further supports transport by Cl^−^/OH^−^ antiport, rather than a cation dependent Na^+^/H^+^ antiport process, due to electrostatic repulsion at the surface of the vesicle with the incoming anion.

**Fig. 3:**
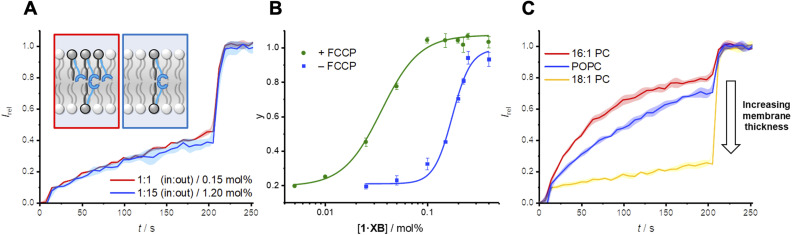
(A) Asymmetric loading of 1·XB to the inner and outer leaflets of POPC LUVs. Symmetric loading was achieved by pre-incorporation of 0.15 mol% 1·XB during LUV preparation (red data). A 15 : 1 excess of 1·XB in the outer leaflet was achieved by addition of a further 1.05 mol% 1·XB in DMSO to pre-formed vesicles loaded with 0.15 mol% 1·XB (blue data, >95% incorporation efficiency). Assay conditions as in Fig. 2. (B) Dependence of fractional activities (*y*, the relative intensity immediately prior to lysis) on concentration of 1middotXB in the presence (green) and absence (blue) of the protonophore FCCP, and fit to the Hill equation (green and blue solid lines, respectively). (C) Dependence of transport activity of 1·XB (0.2 mol% to lipid) on lipid membrane thickness. Assay conditions as in Fig. 2 using the lipid indicated.

The previously discussed kinetics analysis indicates that the anion exchange step between transporters in opposite leaflets is rate-limiting. This presumably proceeds *via* a transient 2 : 1 transporter–anion complex in the membrane interior (Fig. 1A). To probe whether the breaking or formation of this complex is rate-limiting we prepared an asymmetric distribution of relay transporters across the bilayer. This was achieved by externally adding 1.05 mol% 1·XB in DMSO to a suspension of POPC LUVs with 0.15 mol% 1·XB pre-incorporated during preparation, resulting in an excess of relay transporters immobilised in the outer leaflet compared to the inner leaflet (15 : 1 out : in, 1.2 mol% with respect to lipid in total). With 15 times the number of transporters immobilised in the outer leaflet no change in transport activity was observed compared to a 1 : 1 distribution (Fig. 3A). Firstly, this is consistent with our hypothesis that the inter-relay exchange step within the membrane is rate-limiting, and not anion binding at the interface, the latter of which would be expected to increase with transporter concentration in the outer membrane leaflet. Secondly, it implies that dissociation of the 2 : 1 transmembrane anion complex is rate-limiting, again because an increase in receptors in the outer leaflet would be expected to enhance the rate of formation of the transmembrane complex. We have previously observed the same effect with a HB thiourea relay,^42^ and this can be rationalised by considering that the low polarity environment of the membrane interior will enhance ion association to the receptors.^49,50^

### Origin of Cl^−^ > OH^−^ selectivity in relay and mobile carrier mechanisms

Determination of the transport activity in the presence and absence of the protonophore carbonyl cyanide-*p*-trifluoromethoxyphenylhydrazone (FCCP) was used to quantify the Cl^−^ > OH^−^ anion transport selectivity of 1·XB (Fig. 3B). In this assay, the protonophore mediates fast electrogenic H^+^ transport, uncoupling the H^+^/OH^−^ transport from that of Cl^−^ such that the assay reports on the now rate limiting Cl^−^ transport. The relative selectivity for Cl^−^ > H^+^/OH^−^, *F*(Cl^−^/OH^−^), is given by the ratio of initial rate constants, *k*_ini_(Cl^−^)/*k*_ini_(OH^−^), in the absence and presence of FCCP under the assay conditions. For both triazole-derived relay transporters 1·XB and 1·HB, given the probable Cl^−^/OH^−^ antiport mechanism, the assay will report on Cl^−^ > OH^−^ selectivity.

The observed anion transport by 1·HB was independent of the presence of FCCP, indicating no selectivity for Cl^−^ > OH^−^ (*F* = 1) in line with previous results for unselective prototriazole mobile carriers.^22,32^ In contrast, appreciable Cl^−^ > OH^−^ selectivity was observed for halogen bonding derivative 1·XB (*F* = 12). In the presence of FCCP, the rate-limiting step of Cl^−^ transport was similarly determined to be bimolecular *via* initial rates analysis (Fig. S32†). A similar analysis conducted by determining the ratio of EC_50_ values in the absence and presence of FCCP provided an alternative measure of the Cl^−^ > H^+^/OH^−^ selectivity, *F*′ (where *F*′ = EC_50_/EC_50_^FCCP^) under the same assay conditions (Table 1). This also revealed appreciable Cl^−^ > OH^−^ selectivity for the XB relay system, whilst no such selectivity was observed for the HB analogue. Given that the rate-limiting process is dissociation of the receptor–anion complex in the centre of the membrane, the observed overall rate of transport is therefore related to the product of the dissociation rate constant, *k*_d_, and the concentration of the 2 : 1 relay-anion (A^−^) complex in the membrane, *k*_d_[1_2_·A^−^]. In contrast to mobile carriers where typically anion complex dissociation is not rate-limiting, it is notable here that the observed transport rate is dependent on dissociation of the complex. We therefore tentatively suggest that the observed preference of 1·XB for Cl^−^ > OH^−^ in the relay transport mechanism in part arises from more facile exchange of chloride between the halogen bonding donors anchored in opposite leaflets, consistent with our previous theoretical calculations which revealed stronger hydroxide binding to iodotriazole derivatives than chloride in apolar solvent.^32^

**Table 1:** Transport activity of halogen bonding (1·XB) and hydrogen bonding (1·HB) relay transporters

Relay transporter in various lipids		*k*_ini_ a(Cl^−^)^FCCP^/10^−3^ s^−1^	*k*_ini_ b(OH^−^)/10^−3^ s^−1^	*F* c(Cl^−^/OH^−^)	EC_50_^d^/mol%	EC_50_^FCCP^^d^/mol%	*F*′(Cl^−^/OH^−^)^e^
1·XB	16 : 1 PC	82(2)	20.8(0.1)	4.0	—	—	—
	POPC	64(2)	5.2(0.1)	12	0.18(0.01)	0.036(0.002)	5.0
	18 : 1 PC	22(1)	0.9(0.1)	24	—	—	—
1·HB	POPC	3.7(0.1)	3.6(0.2)	1.0	0.58(0.03)	0.49(0.02)	1.2

a Initial rates of chloride transport (*k*_ini_) obtained using the HPTS assay for each transporter in the presence of FCCP (0.8 mol%).

b Initial rates of hydroxide transport (*k*_ini_) obtained using the HPTS assay for each transporter.

c Factor of enhancement in the transport rate between Cl^−^ and OH^−^ (*F*(Cl^−^/OH^−^) = *k*_ini_(Cl^−^)/*k*_ini_(OH^−^)) determined using the FCCP assay.

d EC_50_ is defined as the effective concentration required to achieve 50% activity in the presence or absence of FCCP (0.8 mol% with respect to lipid).

e Factor of enhancement in the transport rate between Cl^−^ and OH^−^ (*F*′(Cl^−^/OH^−^) = EC_50_/EC_50_^FCCP^). Initial rates were determined for 1·XB at 0.2 mol% and at 0.6 mol% for 1·HB. Errors at the 95% confidence limit.

A strong dependence of transport rates on lipid bilayer thickness is characteristic of relay transport.^41,42^ With relay 1·XB, we observed a decrease in rate with increasing bilayer thickness whilst maintaining a constant phosphocholine head group, going from 1,2-dipalmitoleoyl-*sn*-glycero-3-phosphocholine (16 : 1 PC), to POPC, and finally 1,2-dioleoyl-*sn*-glycero-3-phosphocholine (18 : 1 PC) (Fig. 3C). Cl^−^ > OH^−^ anion selectivity was also maintained across all three membranes. This strong rate dependence on membrane thickness serves as further evidence that the exchange step of relay transport is rate-limiting, as this step would be expected to be markedly affected by a change in the thickness of the hydrophobic region of the bilayer.

**Fig. 4:**
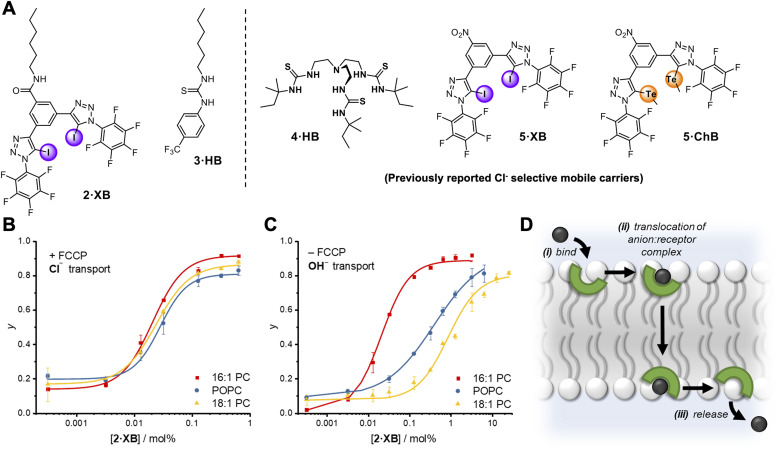
(A) Mobile carrier analogue 2·XB, hydrogen bonding control 3·HB; and known chloride-selective anionophores 4·HB, 5·XB and 5·ChB exploiting hydrogen, halogen and chalcogen bonding interactions, respectively. (B and C) Dependence of fractional activities (*y*, the relative intensity immediately prior to lysis) on concentration of 2·XB and lipid thickness in the presence (C) and absence (D) of FCCP, and fit to the Hill equation (lipid denoted by different colours). Assay conditions as in Fig. 3 using the lipid indicated. (D) Schematic representation of the mobile carrier mechanism with key steps labelled.

The relay transport mechanism involves rate-limiting transfer of the anion between transporters within the membrane interior. In contrast, the rate of ion transport mediated by mobile carriers is typically dominated by the interfacial binding rate (*i.e.* the product of ion association rate, *k*_a_, and transporter concentration, *k*_a_⋅[transporter], for a given ion concentration) when not under saturation conditions where all carriers are complexed.^1^ This leads to a strong dependence of anion transport activity on carrier-anion affinity, and the nature of the lipid headgroup.^51^ However, the effect of membrane thickness has not to our knowledge been investigated for mobile carrier mediated anion transport.^52^ We therefore sought to compare the anion transport properties of relay 1·XB with an analogous non-anchored mobile carrier, to delineate the effect on anion transport of anchoring the XB anion receptor to a lipid scaffold. To this end, a mobile carrier featuring the same XB donor motif, 2·XB, was also prepared (Fig. 4A). In addition, a benchmark unselective HB thiourea based transporter, 3·HB^53^ was synthesised along with three previously reported Cl^−^ selective mobile carriers, 4·HB,^14,54^5·XB^32^ and 5·ChB,^32^ to probe the effect of membrane thickness on mobile carrier transport activity and selectivity. ^1^H NMR anion binding titration experiments with 2·XB and chloride confirmed that this bidentate halogen bonding receptor is capable of strong 1 : 1 anion binding (*K*_a_ = 4660 M^−1^) in a competitive organic-aqueous solvent mixture of acetone-d_6_ : D_2_O (v/v 97.5/2.5).

Mobile carrier 2·XB proved to be an effective anion transporter (EC_50_ = 0.028 mol%, Table 2). As with the relay, appreciable Cl^−^ > OH^−^ selectivity (*F*′ = 14) in POPC LUVs was also observed (Fig. 4B and C), while the HB thiourea 3·HB exhibits no selectivity (Fig. S38†), indicative of rate-limiting chloride transport. We also explored the rates of both chloride and hydroxide transport mediated by 2·XB and 3·HB across a range of lipid membranes of varying depths with identical head groups (16 : 1 PC, POPC and 18 : 1 PC, Fig. 4B and C, and Table 2). In the case of transport experiments which report on rate-limiting chloride transport (*i.e.*2·XB with FCCP, and 3·HB without FCCP), no dependence on lipid thickness was observed (Fig. 4B and S39–S42†). This indicates that under these experimental conditions the rate of interfacial chloride binding is slow compared to the rate of translocation of the chloride-carrier complex. Surprisingly however, hydroxide transport by 2·XB (reported on by conducting the transport assays in the absence of FCCP), exhibited a strong dependence on lipid membrane thickness in which activity decreases with increasing lipid chain length (Fig. 4C). Hill analysis of the dose–response curves revealed increasing Cl^−^ > OH^−^ selectivity factors *F*′ with increasing membrane thickness. The overall rate of anion transport is a function of both interfacial binding rate and translocation. The membranes used differ only in length of the phospholipid tails and have identical headgroups, and so are expected to have near identical interfacial anion binding rates. The implication is therefore that the different rates of membrane translocation of the chloride and hydroxide complexes of 2·XB are responsible for the observed selectivity for chloride in the thicker membranes (step ii, Fig. 4D), and not binding selectivity at the interface (step i).

**Table 2:** Transport activity of halogen bonding (2·XB) and hydrogen bonding (3·HB) mobile carriers

Mobile carrier in various lipids		EC_50_^a^/mol%	EC_50_^FCCP^^a^/mol%	*F*′(Cl^−^/OH^−^)^b^
2·XB	16 : 1 PC	0.021(0.001)	0.021(0.001)	1.0
	POPC	0.40(0.09)	0.028(0.006)	14
	18 : 1 PC	0.9(0.1)	0.029(0.002)	31
3·HB	16 : 1 PC	0.22(0.01)	—	—
	POPC	0.23(0.01)	—	—
	18 : 1 PC	0.31(0.01)	—	—

a EC_50_ defined as the effective concentration needed for 50% activity at *t* = 276 s, in the presence or absence of FCCP; values reported in transporter to lipid molar ratio (mol%).

b Factor of enhancement in the transport rate between Cl^−^ and OH^−^ (*F*′(Cl^−^/OH^−^) = *EC*_50_/*EC*_50^FCCP^_) using the FCCP assay. Errors at the 95% confidence limit.

These results suggest that in the case of hydroxide transport mediated by the XB carrier 2·XB, it is the comparatively slow rate of translocation of the 1 : 1 hydroxide-carrier complex through the hydrophobic region of the bilayer which dominates the overall transport rate in the thicker 18 : 1 PC and POPC membranes. In contrast, for the thinnest membrane (16 : 1 PC) the Cl^−^ > OH^−^ selectivity is lost, suggesting that the translocation of the hydroxide-carrier complex in this case is now comparatively fast compared to interfacial binding. We postulate that this may be due to an increasing activation barrier for translocation of the hydrophilic OH^−^-2·XB complex through the membrane interior, as the hydrophobic region of the bilayer increases. In contrast, this barrier is diminished for the less hydrophilic chloride anion complex of 2·XB, as well as by the benchmark chloride anionophore 3·HB. In comparison, for the relay transporters, the rate-limiting step is the anion exchange step, and thus selectivity is a function of the relative ease of dissociation of the transmembrane 2 : 1 relay-anion complex within the centre of the membrane.

To explore whether chloride selectivity of previously reported selective anionophores is also dependent on membrane thickness, and hence arises from differing rates of membrane translocation of the chloride and hydroxide/proton complexes, we explored the transport rates of the two anions with 4·HB, 5·XB and 5·ChB in the three membranes of varying thickness previously described (Table 3). In each case, as with 2·XB, the rate of chloride transport (in the presence of FCCP) was invariant with membrane thickness, whilst that of hydroxide transport decreased with increasing lipid length, resulting in increasing Cl^−^ > OH^−^ selectivity as the membrane thickness increases. This suggests that across all four chloride-selective carriers studied which span a range of structures, anion binding groups and intermolecular interactions, the observed Cl^−^ > OH^−^ selectivity arises from differing rates of membrane translocation of the chloride and hydroxide complexes.

**Table 3:** Rates analysis of transport activity for a range of mobile carriers (2·XB, 3·HB, 4·HB, 5·XB and 5·ChB) in the presence and absence of FCCP, in different lipid membranes

Mobile Carrier in various lipids	*k*_ini_(Cl^−^)^FCCP^^a^/10^−3^ s^−1^			*k*_ini_(OH^−^)^b^/10^−3^ s^−1^		
	16 : 1 PC	POPC	18 : 1 PC	16 : 1 PC	POPC	18 : 1 PC
2·XB^c^	12.3(0.1)	10.0(0.6)	10.4(0.1)	12(1)	2.5(0.1)	1(0.1)
3·HB^d^	6.2(0.4)	5.8(0.9)	7.5(0.5)	—	5.8(0.9)	—
4·HB^e^	2.6(0.3)	3.6(0.1)	3.7(0.5)	3.6(0.3)	11(5)	1.3(0.1)
5·XB^f^	4.7(0.1)	3.0(0.2)	2.7(0.1)	3.3(0.2)	2.1(0.1)	1.8(0.5)
5·ChB^g^	2.1 (0.1)	2.2 (0.4)	2.2(0.1)	4.3(0.4)	2.5(0.1)	3.3(0.2)

a Initial rates of chloride transport (*k*_ini_) obtained using HPTS assay for each transporter in the presence of FCCP (0.8 mol%).

b Initial rates of hydroxide transport (*k*_ini_) obtained using HPTS assay for each transporter.

c 2·XB rates analysis at 0.128 mol%.

d 3·HB rates analysis at 0.32 mol%.

e 4·HB rates analysis at 0.0032 mol% with FCCP and 0.016 mol% without FCCP.

f 5·XB rates analysis at 0.0032 mol% with FCCP and 0.016 mol% without FCCP.

g 5·ChB rates analysis at 0.01 mol% with FCCP and 0.1 mol% without FCCP. Errors at the 95% confidence limit.

The dependence of anion selectivity on lipid bilayer thickness has not, to the best of our knowledge, been previously studied and these results suggest that considering the relative rate of transport of different ions across a membrane will be critical to the design of selective transporters in the future. Importantly, for the application of synthetic anionophores as clinically relevant therapeutics, high anion selectivity is key (particularly Cl^−^ > H^+^/OH^−^). The dependence of their ion selectivity on the complex mixture of lipids present in cellular membranes must therefore be carefully considered.

## Conclusions

We report the first example of a halogen bonding (XB) membrane-anchored ion carrier, with record activity for a transporter which operates *via* a relay mechanism. Analysis of the transport rates and using asymmetric distributions of relay transporters in the membrane reveals that anion exchange between transporters in the membrane interior is rate-limiting, and faster for chloride than hydroxide for the XB relay 1·XB, leading to a high selectivity for Cl^−^ > OH^−^. The strong dependence of transport rate for both chloride and hydroxide with membrane thickness is consistent with a relay mechanism. Comparison with an analogous XB mobile carrier 2·XB revealed no dependence on membrane thickness for chloride transport, as observed for a typical thiourea-based HB anionophore 3·HB. A strong membrane thickness dependence for hydroxide transport rates with 2·XB, as well as for a family of other known chloride-selective non-protonophoric carriers, was observed with a concomitant dependence on Cl^−^/OH^−^ selectivity. These results reveal that changing membrane thickness, whilst maintaining identical lipid headgroups, leads to significant modulation of Cl^−^ > OH^−^ selectivity. This serves to modulate membrane translocation rates of the anion–carrier complex, and this effect can dominate over anion binding at the membrane–aqueous interface. In contrast, the XB relay ionophore showed no such membrane thickness dependence, maintaining the desired Cl^−^ > OH^−^ selectivity. We anticipate that these novel mechanistic insights into the properties governing anion selectivity for both mobile and membrane-anchored carriers will provide a basis for the design of selective anionophores for future therapeutic applications, and also highlight the unique properties of relay transporters over their mobile carrier counterparts.

## Author contributions

T. G. J., A. D. and A. S.-K. synthesised the compounds and conducted the ion transport assays. M. J. L. conceived and supervised the project. All authors contributed to data analysis and writing of the manuscript.

## Conflicts of interest

There are no conflicts to declare.

## Supplementary Material

SC-014-D3SC01170D-s001
