# Supplementary material for: Halogen bonding relay and mobile anion transporters with kinetically controlled chloride selectivity
Source: Chem Sci. 2023 Apr 4;14(19):5006–13. doi: 10.1039/d3sc01170d (PMC10189858; doi:10.1039/d3sc01170d)
Supplement: SC-014-D3SC01170D-s001 [file SC-014-D3SC01170D-s001.pdf]

Electronic Supplementary Material (ESI) for Chemical Science.
This journal is © The Royal Society of Chemistry 2023

Supporting Information for

Halogen bonding relay and mobile anion transporters with kinetically controlled chloride selectivity

Toby G. Johnson, Andrew Docker, Amir Sadeghi-Kelishadi and Matthew J. Langton*
Chemistry Research Laboratory, University of Oxford, Mansfield Road, Oxford, OX1 3TA, UK

Contents

1 Materials and methods 2
2 Synthesis and characterization 3
3 UV-Visible Absorption Analysis 20
4 Anion Transport Experiments 22
5 ^{1}H NMR Titration Experiments 35
6 References 36

1 Materials and methods

All reagents and solvents were purchased from commercial sources and used without further purification. Lipids were purchased from Avanti polar lipids and used without further purification. Where necessary, solvents were dried by passing through an MBraun MPSP-800 column and degassed with nitrogen. Triethylamine was distilled from and stored over potassium hydroxide. Normal phase silica gel flash column chromatography was performed manually using Merck® silica gel 60 under a positive pressure of nitrogen. Where mixtures of solvents were used, ratios are reported by volume. NMR spectra were recorded on a Bruker AVIII 400, Bruker AVII 500 (with cryoprobe) and Bruker AVIII 500 spectrometers. Chemical shifts are reported as δ values in ppm. Mass spectra were carried out on a Waters Micromass LCT and Bruker microTOF spectrometers. Fluorescence spectroscopic data were recorded using a Horiba Duetta fluorescence spectrophotometer, equipped with a Peltier temperature controller and stirrer. UV-Vis spectra were recorded on a V-770 UV-Visible/NIR Spectrophotometer equipped with a Peltier temperature controller and stirrer, using quartz cuvettes of 1 cm path length. Experiments were conducted at 25 °C unless otherwise stated. Vesicles were prepared as described below using Avestin "LiposoFast" extruder apparatus, equipped with polycarbonate membranes with 200 nm pores. GPC purification of vesicles was carried out using GE Healthcare PD-10 desalting columns prepacked with Sephadex G 25 medium.

Abbreviations

16:0 Lyso PC: 1-Palmitoyl-2-hydroxy-*sn*-glycero-3-phosphocholine; **16:1 PC**: 1,2-Dipalmitoleoyl-*sn*-glycero-3-phosphocholine; **18:1 PC**: 1,2-Dioleoyl-*sn*-glycero-3-phosphocholine (DOPC); **DBU**: 1,8-Diazabicyclo[5.4.0]undec-7-ene; **DIC**: *N*,*N*-Diisopropylcarbodiimide; **DIPEA**: *N,N*-Diisopropylethylamine; **DMAP**: 4-Dimethylaminopyridine; **DPPC**: 1,2-Dipalmitoyl-sn-glycero-3-phosphocholine; **FCCP**: Carbonyl cyanide-p-trifluoromethoxyphenylhydrazone; **Fmoc**: 9-Fluorenylmethoxycarbonyl; **HEPES**: *N*-(2-Hydroxyethyl)piperazine-*N*'-(2-ethanesulfonic acid); **HPTS**: 8-Hydroxy-1,3,6-pyrenetrisulfonate; **HRMS**: High resolution mass spectrometry; **LUVs**: Large unilamellar vesicles; **POPC**: 1-Palmitoyl-2-oleoyl-*sn*-glycero-3-phosphocholine; **POPG**: 1-Palmitoyl-2-oleoyl-*sn*-glycero-3-phospho-(1'-rac-glycerol) sodium salt; **rt**: Room temperature.

2 Synthesis and characterization

2.1 General comments.

Compounds **S1·HB**[2], **S1·XB**[2], **S3**[1], **3·HB**[3], **4·HB**,[4] **5·XB**[5] and **5·ChB**[5] were prepared according to literature procedures.

S2·HB and **S2·XB** were synthesized according the following general procedure:

$[Cu(MeCN)_4]PF_6$ (25 mg, 0.0672 mmol) and TBTA (36 mg, 0.0672 mmol) were dissolved in the minimum amount anhydrous degassed CH_2Cl_2 (ca. 8 ml) and left to stir for 15 minutes. After which time the appropriate alkyne, either **S1·HB** and **S1·XB** (0.336 mmol) was added to the solution as a solid, followed by pentafluorophenyl azide (352 mg, 1.68 mmol). Once complete, as determined by TLC analysis, the reaction mixture was diluted with CH_2Cl_2 (ca. 150 ml) and washed with EDTA/NH_4OH(aq) solution (20 ml) and water (50 ml), the collected organic phase was dried over $MgSO_4$ and concentrated to dryness and the crude residues were purified by silica gel column chromatography (EtOAc:CH_2Cl_2, 2:98, v/v).

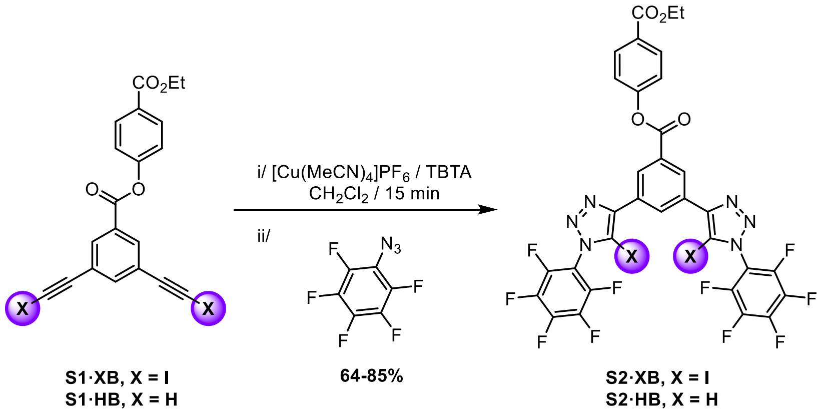

***Scheme S1**. Synthesis of bidentate hydrogen (**S2·HB**) and halogen (**S2·XB**) bonding activated esters.*

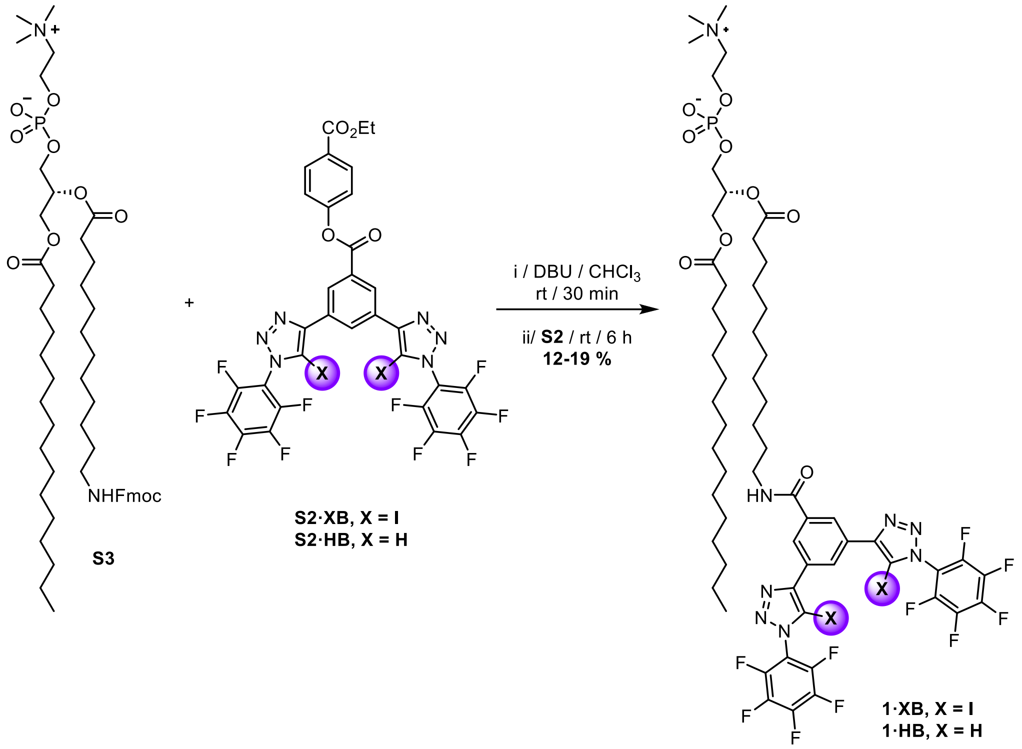

Scheme S2. *Synthesis of hydrogen (**1·HB**) and halogen (**1·XB**) bonding relay transporters.*

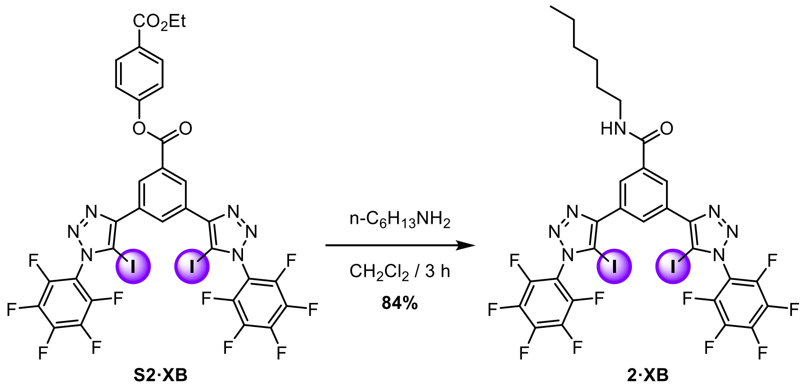

Scheme S3. *Synthesis of halogen bonding mobile carrier **2·XB**.*

2.2 Halogen bonding activated ester, S2·XB

Isolated as a white solid (212 mg, 0.215 mmol, 64%).

^{1}H NMR (500 MHz, $CDCl_3$) δ 9.08 (t, *J* = 1.7 Hz, 1H, H_a), 9.01 (d, *J* = 1.7 Hz, 2H, H_b), 8.16 (d, *J* = 8.8 Hz, 2H, H_c), 7.38 (d, *J* = 8.8 Hz, 2H, H_c), 4.40 (q, *J* = 7.1 Hz, 2H, H_e), 1.41 (t, *J* = 7.1 Hz, 3H, H_f).

^{19}F NMR (470 MHz, $CDCl_3$) δ -141.51 – -142.81 (m), -146.76, -158.81 (dd, *J* = 21.0, 16.3 Hz).

^{13}C NMR (126 MHz, $CDCl_3$) δ 165.9, 163.9, 154.5, 149.2, 143.8 (dm, *J* = 260 Hz), 138.2 (dm, *J* = 260 Hz), 131.4, 130.9 (t, *J* = 15.0 Hz), 129.5, 128.6, 121.8, 112.3 (d, *J* = 16.9 Hz), 81.2, 61.3, 14.5.

HRMS-ESI (m/z) Calculated for $C_{32}H_{13}F_{10}O_4N_6I_2$ $[M+H]^+$, 988.8923; found 988.8938.

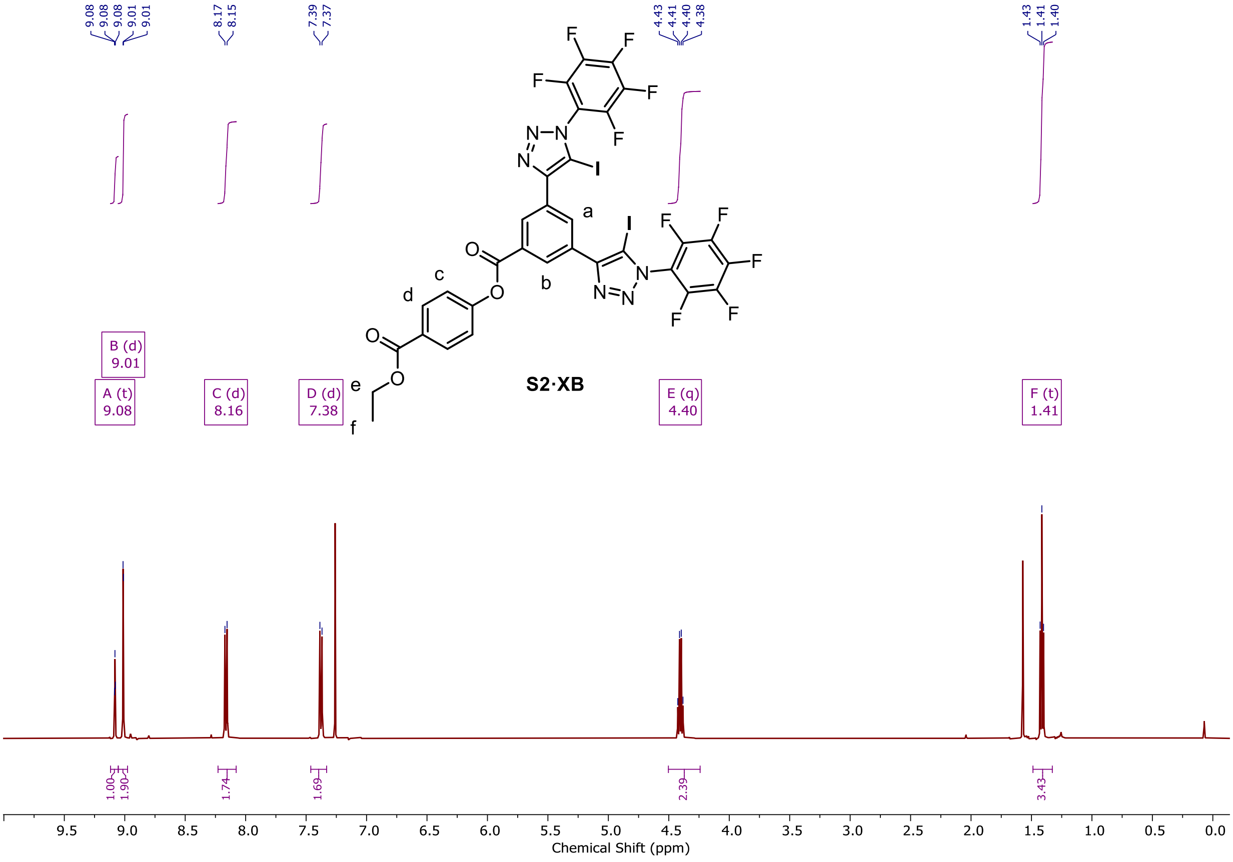

Figure S1. *^{1}H NMR spectrum of compound **S2·XB** ($CDCl_3$, 500 MHz, 298 K).*

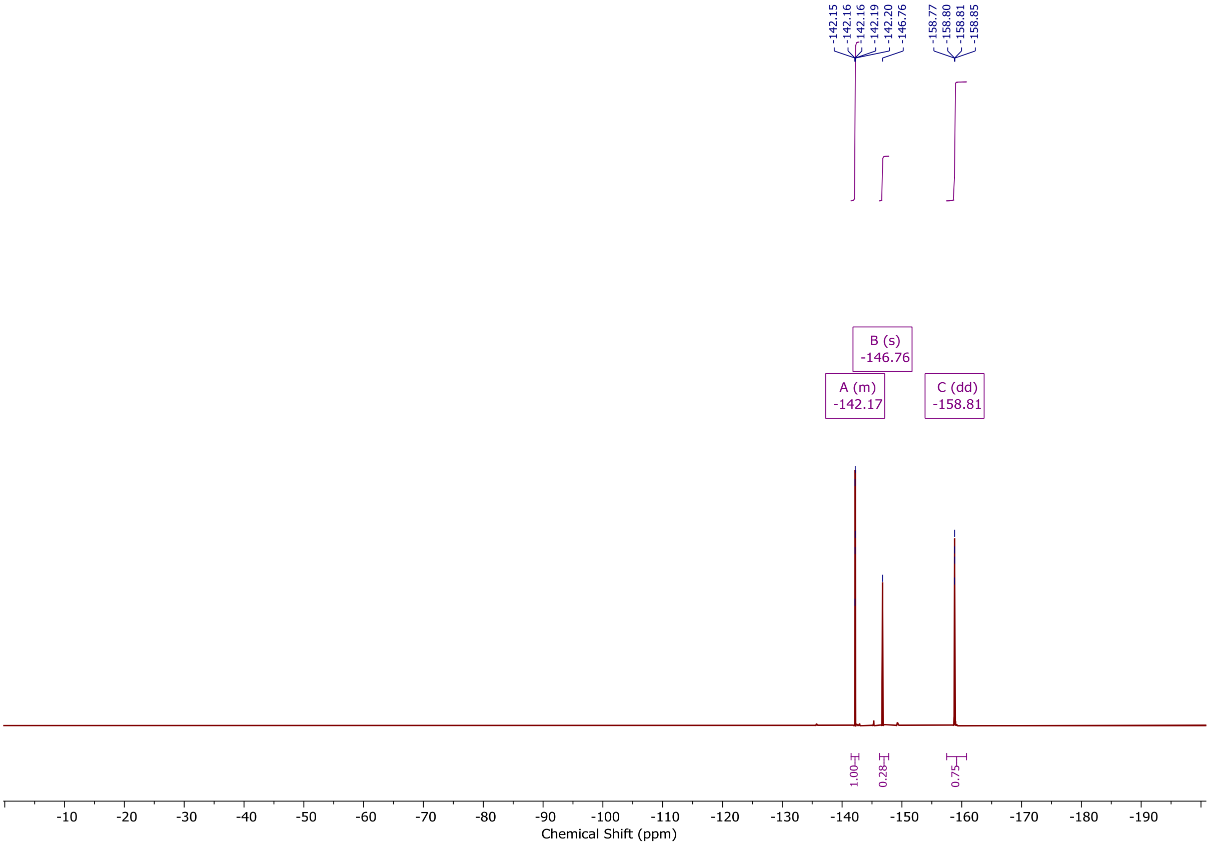

Figure S2. *^{19}F NMR spectrum of compound* ***S2·XB*** *(CDCl₃, 470 MHz, 298 K).*

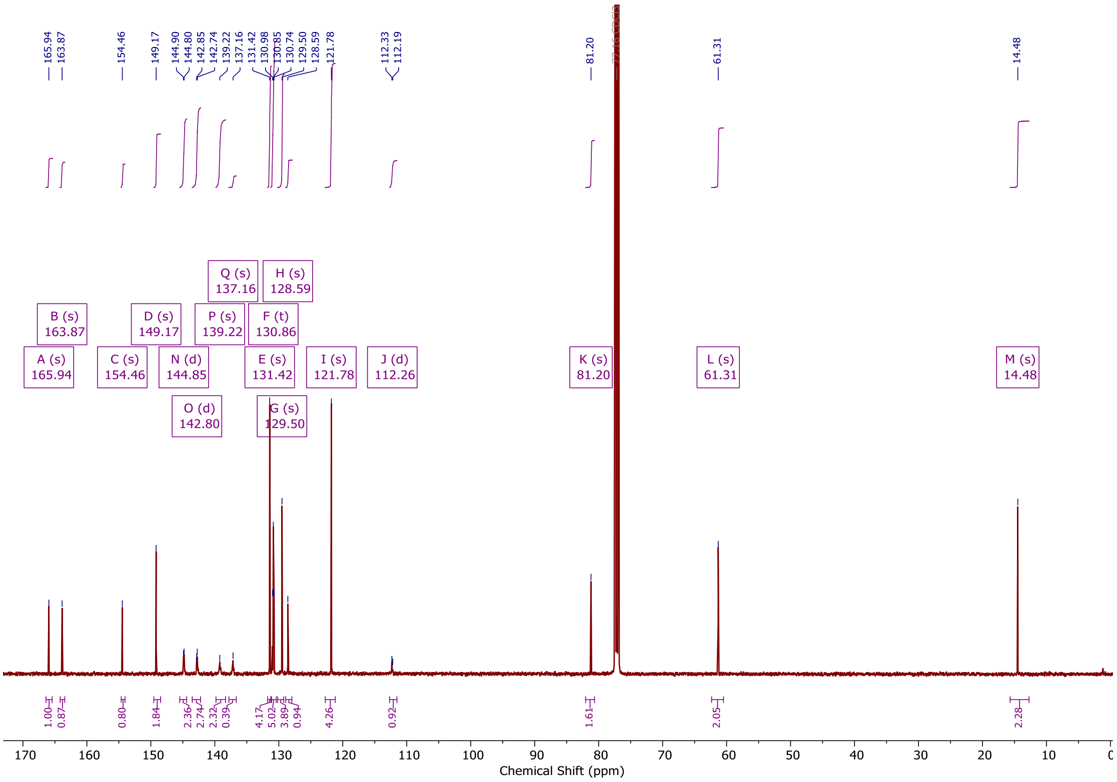

Figure S3. *^{13}C NMR spectrum of compound* ***S2·XB*** *(CDCl₃, 126 MHz, 298 K).*

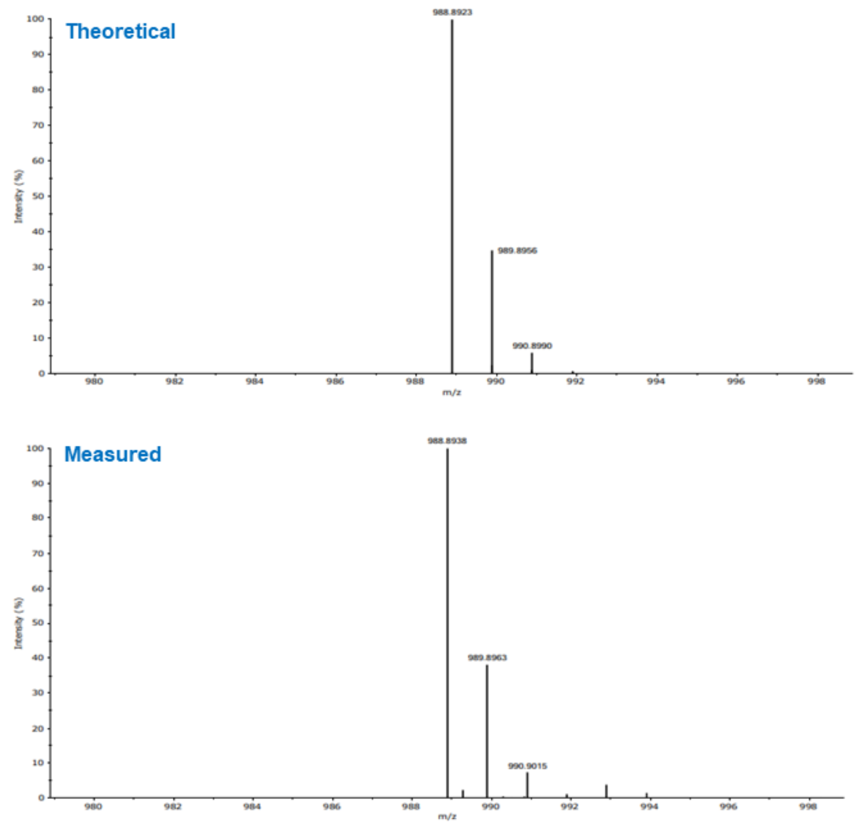

Figure S4. *HRMS spectrum of compound* ***S2·XB****.*

2.3 Hydrogen bonding activated ester, S2·HB

Isolated as a white solid (208 mg, 0.282 mmol, 85%).

^{1}H NMR (400 MHz, $CDCl_3$) δ 8.83 (t, *J* = 1.7 Hz, 1H, H_a), 8.74 (d, *J* = 1.7 Hz, 2H, H_b), 8.29 (s, 2H, H_g), 8.24 – 8.13 (m, 2H, H_d), 7.40 – 7.31 (m, 2H, H_c), 4.41 (q, *J* = 7.1 Hz, 2H, H_e), 1.42 (t, *J* = 7.1 Hz, 3H, H_f).

^{19}F NMR (377 MHz, Acetone-d_6) δ -146.61 – -150.49 (m), -153.20, -160.90 – -164.83 (m).

^{13}C NMR (101 MHz, Acetone-d_6) δ 166.0, 164.6, 155.6, 147.2, 143.5 (dm, *J* = 280 Hz), 139.2 (dm, 255 Hz), 132.6, 132.0, 131.8, 129.3, 128.6, 127.8, 125.6, 123.0, 114.0 (m), 61.7, 14.6. (Some signals coincident and/or non-observable due to C-F coupling.)

HRMS-ESI (m/z) Calculated for $C_{32}H_{15}F_{10}O_4N_6$ $[M+H]^+$, 737.0990; found 737.1011.

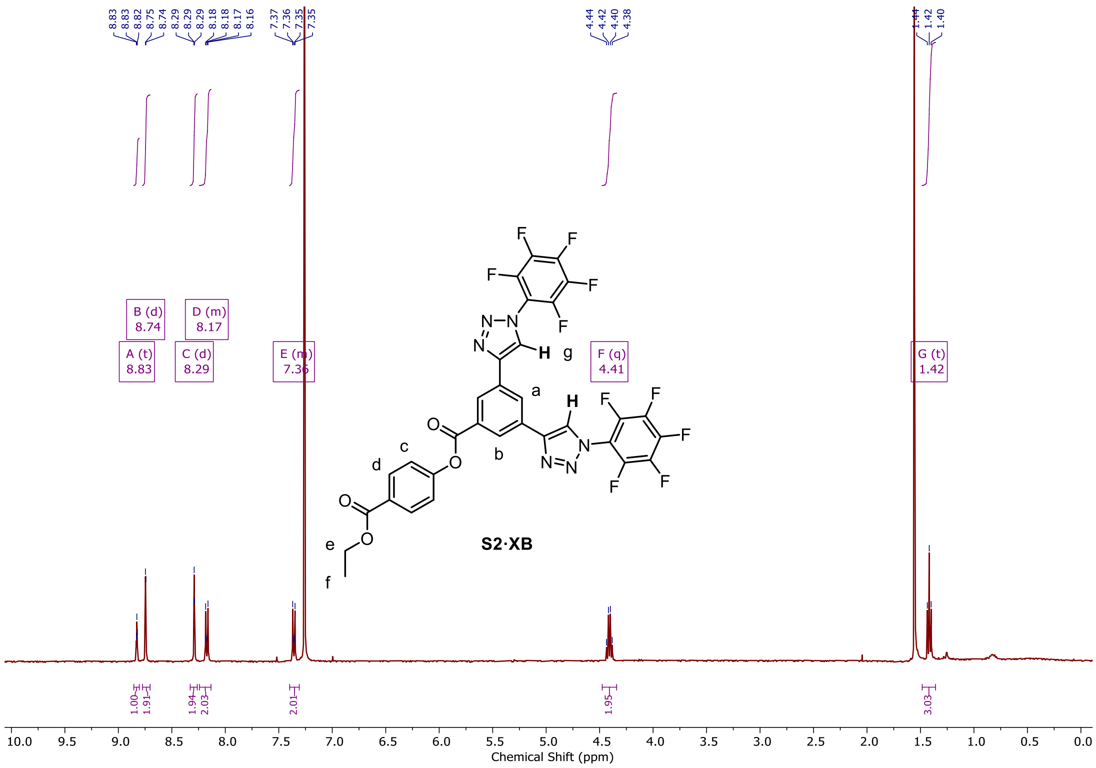

Figure S5. *^{1}H NMR spectrum of compound **S2·HB** (CDCl$_3$, 400 MHz, 298 K).*

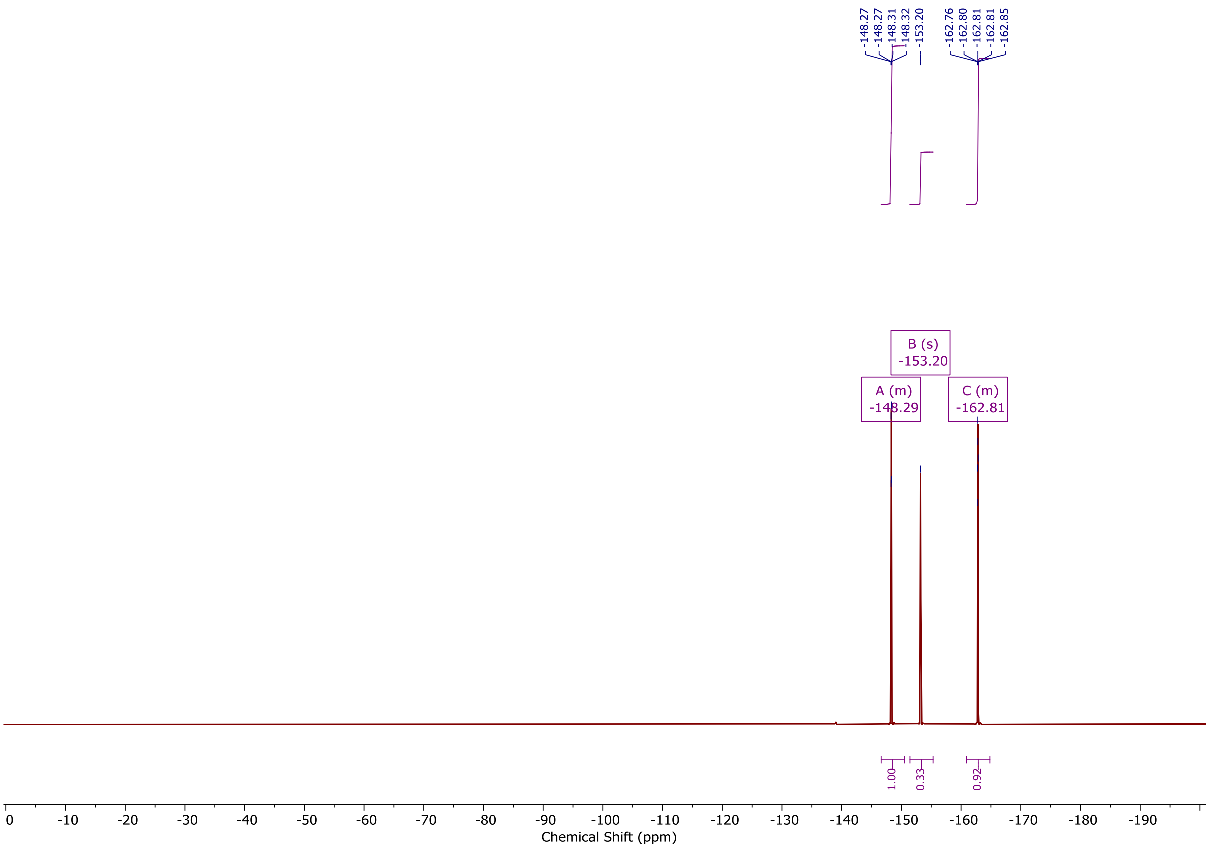

Figure S6. *^{19}F NMR spectrum of compound **S2·HB** (Acetone-d$_6$, 377 MHz, 298 K).*

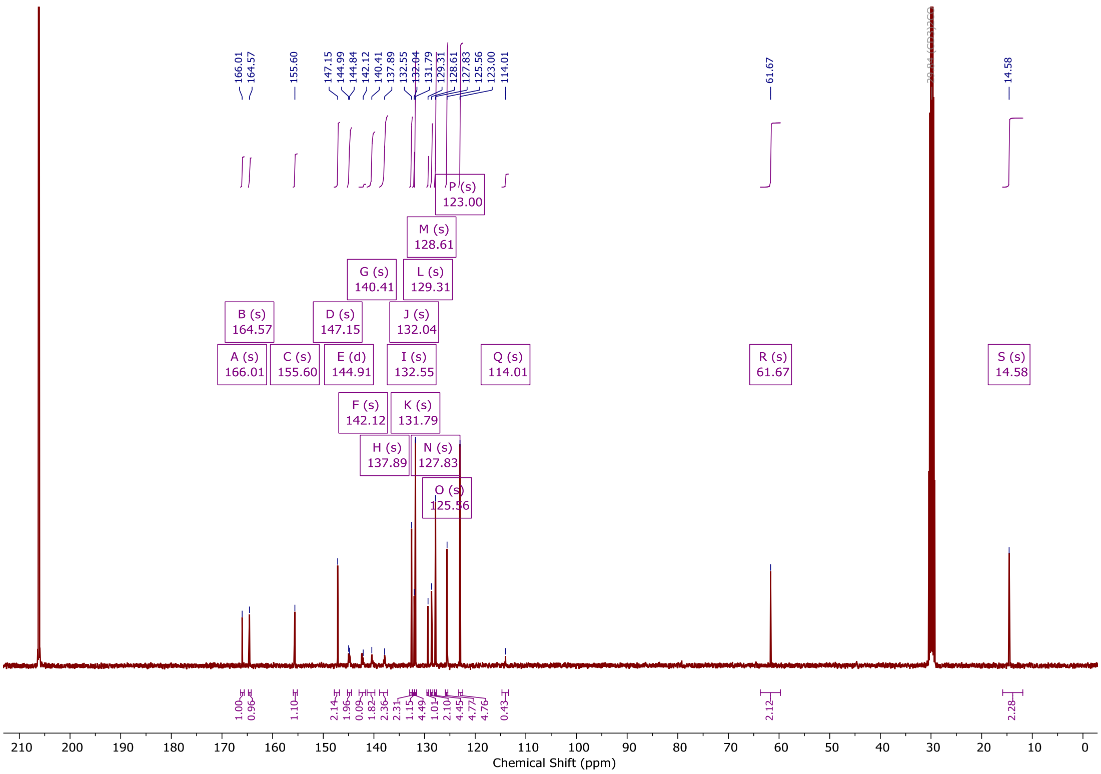

Figure S7. ^{13}C NMR spectrum of compound ***S2·HB*** (Acetone-d_6, 101 MHz, 298 K).

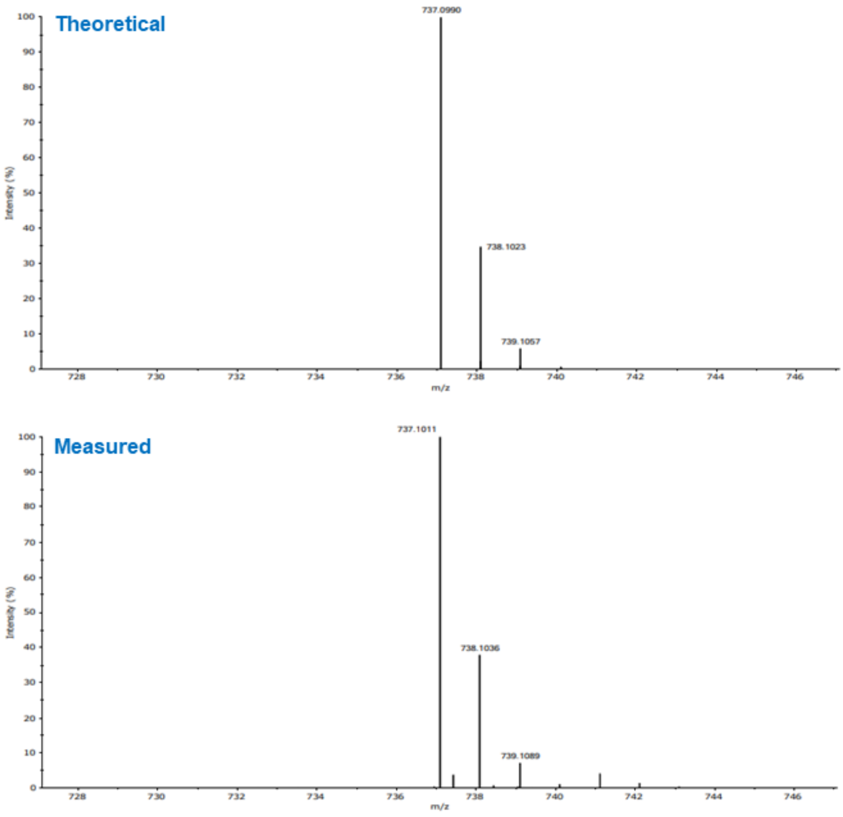

Figure S8. HRMS spectrum of compound ***S2·HB***.

2.4 Halogen bonding relay transporter, 1·XB

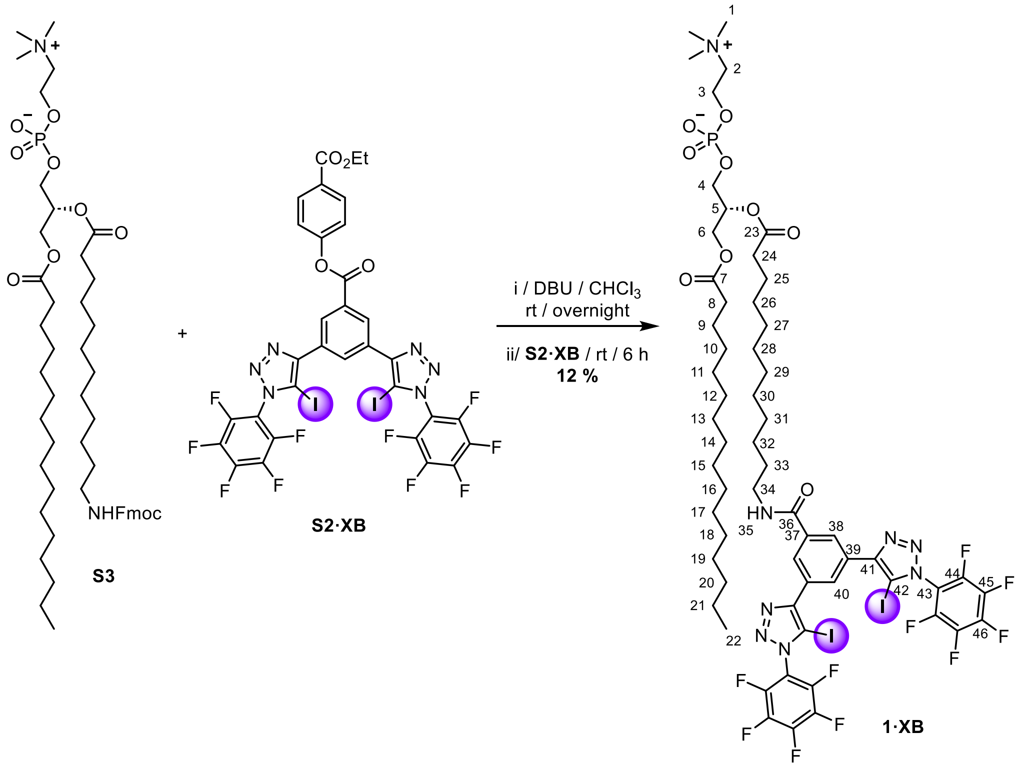

DBU (10 µL, 0.07 mmol, 2 Equiv.) was added to a stirred solution of 12-(Fmoc-amino)dodecanoic acid **S3** (30 mg, 0.03 mmol, 1 Equiv.) in anhydrous $CHCl_3$ (0.25 mL) under N_2. After stirring the reaction mixture overnight, **S2·XB** (65 mg, 0.07 mmol, 2 Equiv.) was added and left to stir for 6 h. The reaction mixture was concentrated and loaded onto a short plug of DOWEX (2 g) and washed with $CHCl_3$:MeOH (1:1, 50 mL) and then flushed off with $CHCl_3$:MeOH:H_2O (65:25:4, 50 mL). This crude product was then purified by prep TLC (1% H_2O / 25% MeOH / $CHCl_3$) to afford the title compound as a white solid (6 mg, 4 µmol, 12%).

^{1}H NMR (600 MHz, $CDCl_3$:CD_3OD (2:1)) δ 8.64 (t, J = 1.4 Hz, 1H, H_{40}), 8.36 (d, J = 1.5 Hz, 2H, H_{38}), 5.05 – 4.97 (m, 1H, H_5), 4.05 – 3.99 (m, 2H, H_3), 3.93 (dd, J = 12.0, 6.9 Hz, 1H, H_6), 3.79 – 3.76 (m, 2H, H_4), 3.40 – 3.37 (m, 2H, H_2), 3.27 – 3.23 (m, 2H, H_{34}), 3.00 (s, 9H, H_1), 2.13 – 2.06 (m, 4H, $H_{8\,\&\,24}$), 1.49 – 1.43 (m, 2H, H_{33}), 1.41 – 1.34 (m, 4H, $H_{9\,\&\,25}$), 1.23 – 1.18 (m, 2H, H_{32}), 1.12 – 1.01 (m, 36H, $H_{10\text{-}21\,\&\,26\text{-}31}$), 0.65 (t, J = 6.9 Hz, 3H, H_{22}). One proton environment for H_6 is obscured by the H_2O peak.

^{13}C NMR (151 MHz, $CDCl_3$:CD_3OD (2:1)) δ 173.8, 173.4, 167.2, 149.4, 144.7 – 142.3 (dm, J = 263 Hz), 138.9 – 136.7 (dm, J = 248 Hz), 136.0, 130.2, 128.1, 126.5, 112.3 – 111.9 (m), 83.2, 70.2 (d, J = 7.8 Hz) (C_5), 66.3 (C_2), 63.4 (d, J = 5.0 Hz) (C_4), 62.4 (C_6), 58.8 (d, J = 4.9 Hz) (C_3), 53.9 (t, J = 3.7 Hz) (C_1), 40.1, 34.0, 33.8, 31.7, 29.4, 29.4, 29.4, 29.4, 29.4, 29.4, 29.3, 29.3, 29.2, 29.2, 29.2, 29.1, 29.1, 29.0, 29.0, 28.9, 28.8, 26.8, 24.6, 24.6, 22.4, 13.6 (C_{22}).

^{19}F NMR (470 MHz, $CDCl_3$:CD_3OD (2:1)) δ -143.20 – -143.38 (m, 2F, F_{44}), -147.88 (t, J = 21.3 Hz, 1F, F_{46}), -159.77 – -159.93 (m, 2F, F_{45}).

HRMS-ESI (m/z) Calculated for $C_{59}H_{76}F_{10}O_9N_8I_2P$ $[M\text{-}H]^-$, 1515.3397; found 1515.3371.

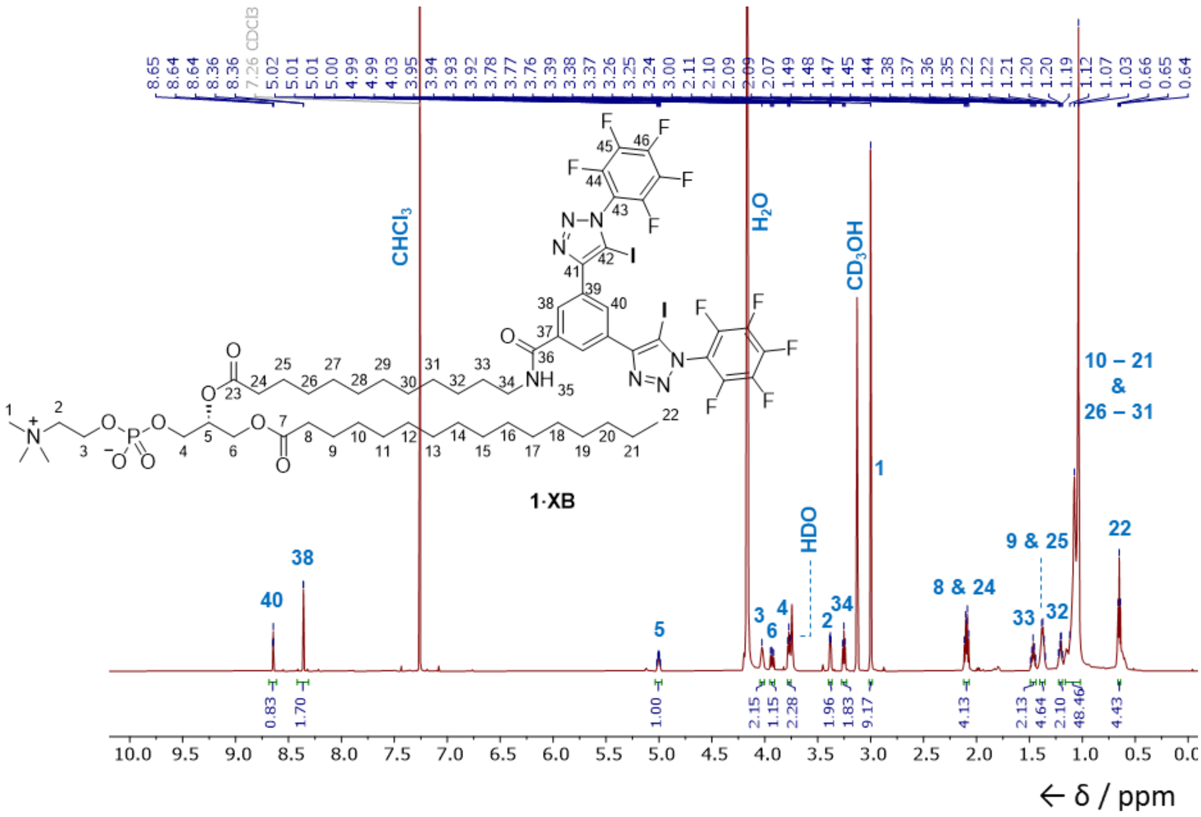

Figure S9. *^{1}H NMR spectrum of compound* ***1·XB*** *($CDCl_3$:CD_3OD (2:1), 600 MHz, 298 K).*

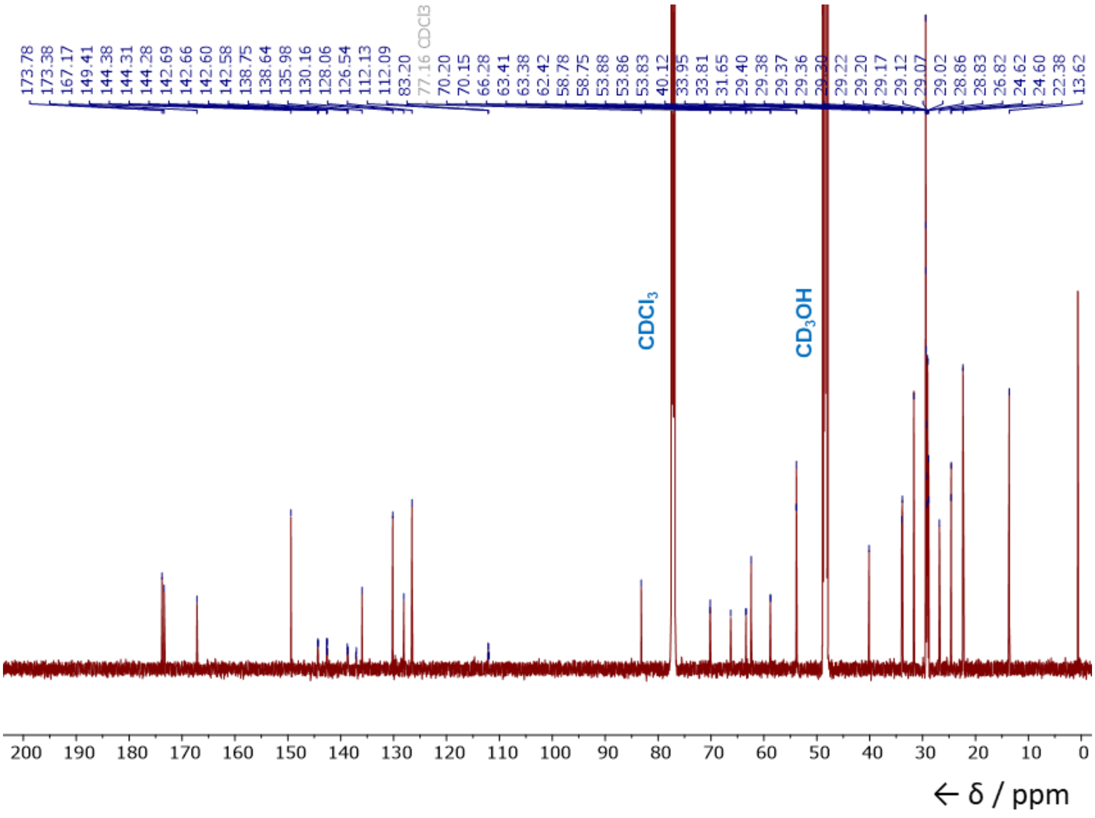

Figure S10. *^{13}C NMR spectrum of compound* ***1·XB*** *($CDCl_3$:CD_3OD (2:1), 151 MHz, 298 K).*

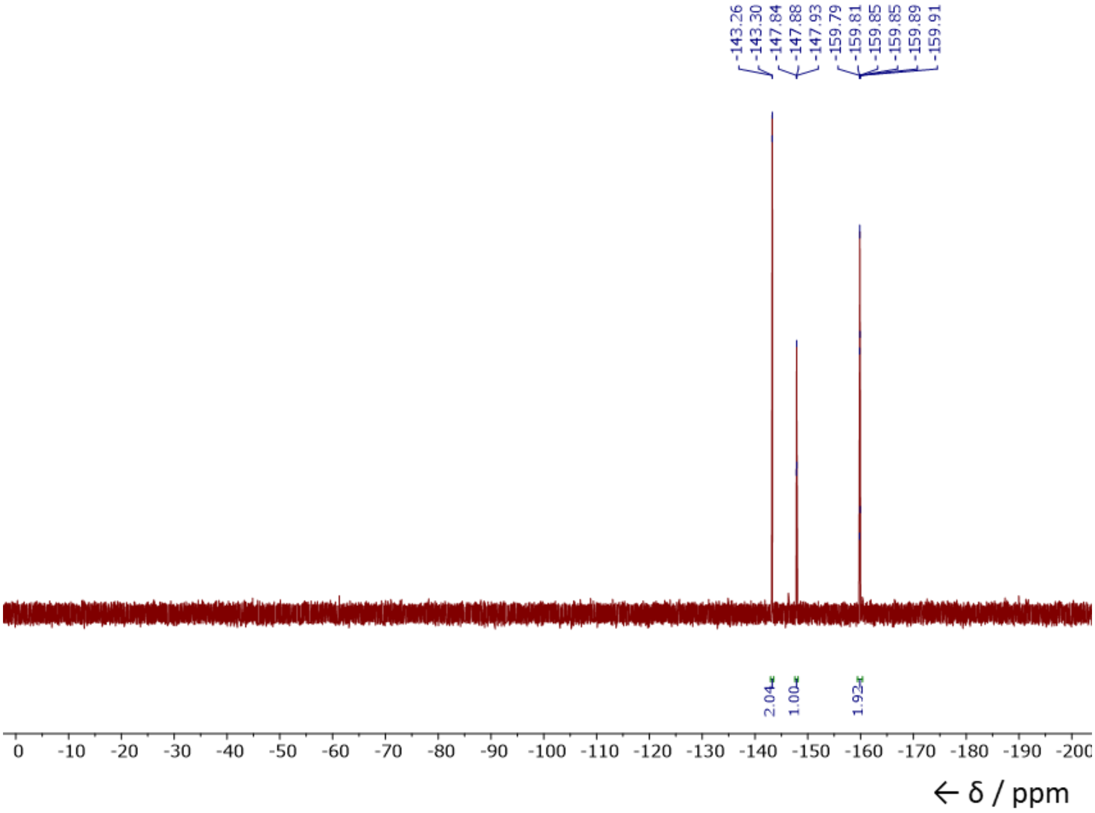

Figure S11. *^{19}F NMR spectrum of compound* ***1·XB*** *($CDCl_3$:CD_3OD (2:1), 470 MHz, 298 K).*

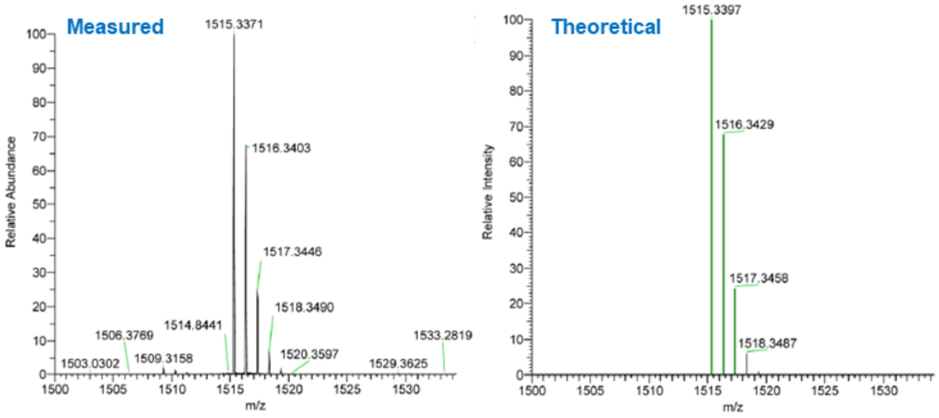

Figure S12. *HRMS spectrum of compound* ***1·XB****. Calculated for $C_{59}H_{76}F_{10}O_9N_8I_2P$ $[M-H]^-$, 1515.3397; found 1515.3371.*

2.5 Hydrogen bonding relay transporter, 1·HB

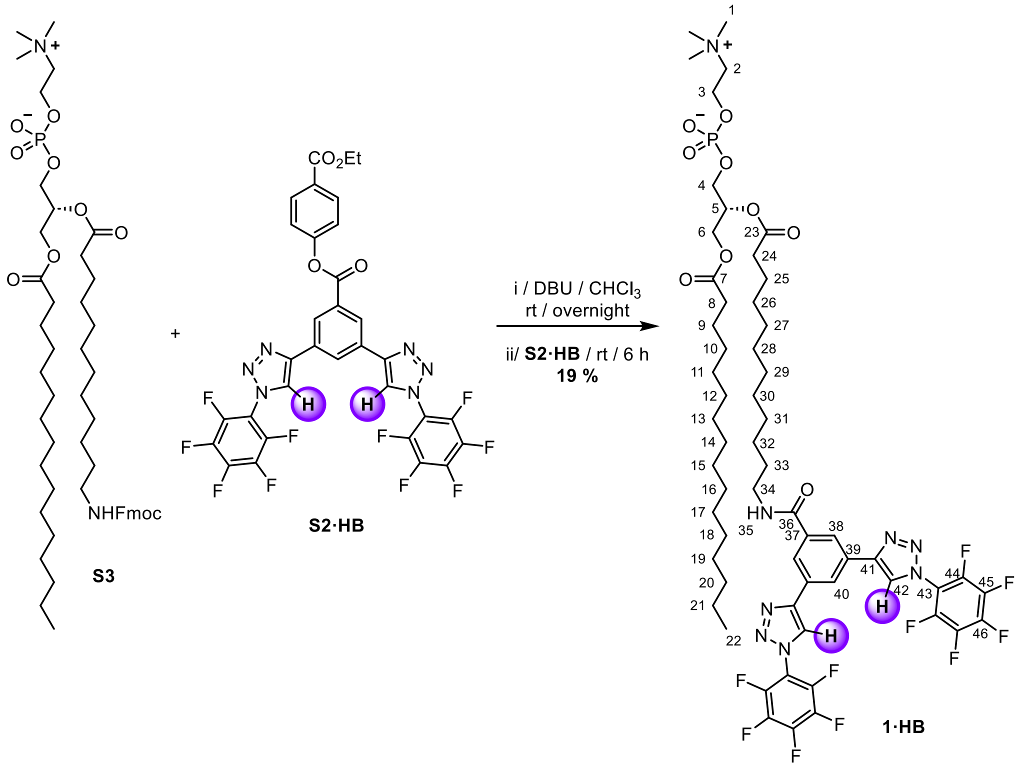

DBU (10 μL, 0.07 mmol, 2 Equiv.) was added to a stirred solution of 12-(Fmoc-amino)dodecanoic acid **S3** (30 mg, 0.03 mmol, 1 Equiv.) in anhydrous $CHCl_3$ (0.25 mL) under N_2. After stirring the reaction mixture overnight, **S2·HB** (50 mg, 0.07 mmol, 2 Equiv.) was added and left to stir for 6 h. The reaction mixture was concentrated and loaded onto a short plug of DOWEX (2 g) and washed with $CHCl_3$:MeOH (1:1, 50 mL) and then flushed off with $CHCl_3$:MeOH:H_2O (65:25:4, 50 mL). This crude product was then purified by prep TLC (1% H_2O / 25% MeOH / $CHCl_3$) to afford the title compound as a white solid (8 mg, 6 μmol, 19%).

^{1}H NMR (500 MHz, $CDCl_3$:CD_3OD (2:1)) δ 8.44 (s, 2H, H_{42}), 8.43 (s, 1H, H_{40}), 8.18 (s, 2H, H_{38}), 7.91 (t, *J* = 5.1 Hz, 1H, H_{35}), 5.04 – 4.95 (m, 1H, H_5), 4.06 – 4.00 (m, 2H, H_3), 3.93 (dd, *J* = 11.9, 6.9 Hz, 1H, H_6), 3.80 – 3.74 (m, 2H, H_4), 3.41 – 3.36 (m, 2H, H_2), 3.28 – 3.22 (m, 2H, H_{34}), 3.00 (s, 9H, H_1), 2.13 – 2.05 (m, 4H, $H_{8\ \&\ 24}$), 1.50 – 1.43 (m, 2H, H_{33}), 1.42 – 1.34 (m, 4H, $H_{9\ \&\ 25}$), 1.24 – 1.17 (m, 2H, H_{32}), 1.13 – 1.01 (m, 36H, $H_{10\text{-}21\ \&\ 26\text{-}31}$), 0.65 (t, *J* = 6.8 Hz, 3H, H_{22}). One proton environment for H_6 is obscured by the H_2O peak.

^{13}C NMR (126 MHz, $CDCl_3$:CD_3OD (2:1)) δ 173.8, 173.4, 167.1, 146.9, 143.5 – 141.2 (dm, *J* = 258 Hz), 139.1 – 136.9 (dm, *J* = 257 Hz), 136.1, 130.4, 125.6, 124.7, 123.6, 112.7 – 112.6 (m), 70.2 (d, *J* = 7.4 Hz) (C_5), 66.3 (C_2), 63.4 (d, *J* = 4.5 Hz) (C_4), 62.4 (C_6), 58.8 (d, *J* = 4.3 Hz) (C_3), 53.9 (t, *J* = 3.6 Hz) (C_1), 40.1 (C_{34}), 33.9, 33.8, 31.6, 29.4, 29.4, 29.4, 29.4, 29.4, 29.4, 29.3, 29.3, 29.2, 29.2, 29.2, 29.1, 29.1, 29.0, 29.0, 28.9, 28.8, 26.8, 24.6, 24.6, 22.4, 13.6 (C_{22}).

^{19}F NMR (470 MHz, $CDCl_3$:CD_3OD (2:1)) δ -146.29 – -146.43 (m, 2F, F_{44}), -150.49 (t, *J* = 21.2 Hz, 1F, F_{46}), -160.20 – -160.35 (m, 2F, F_{45}).

HRMS-ESI (m/z) Calculated for $C_{59}H_{88}F_{10}O_9N_8P$ $[M+H]^+$, 1263.5464; found 1263.5490.

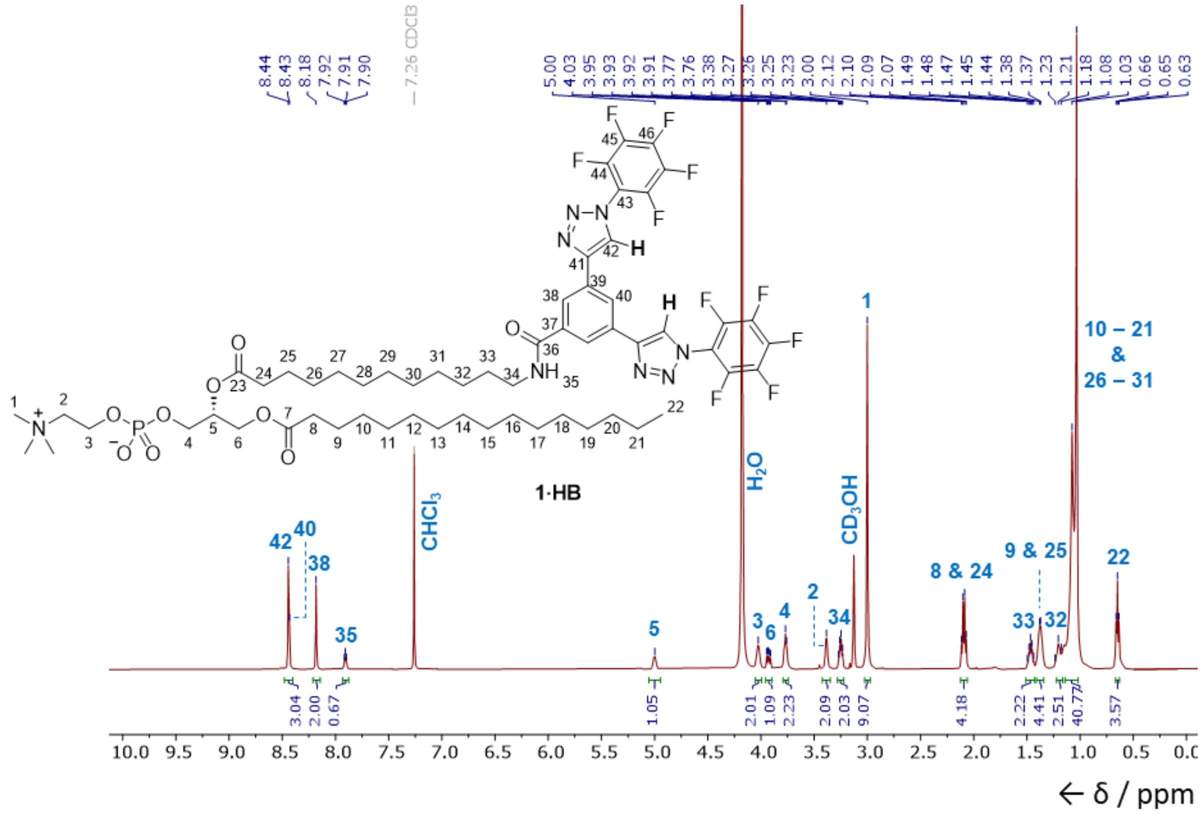

Figure S13. *^{1}H NMR spectrum of compound **1·HB** ($CDCl_3$:CD_3OD (2:1), 500 MHz, 298 K).*

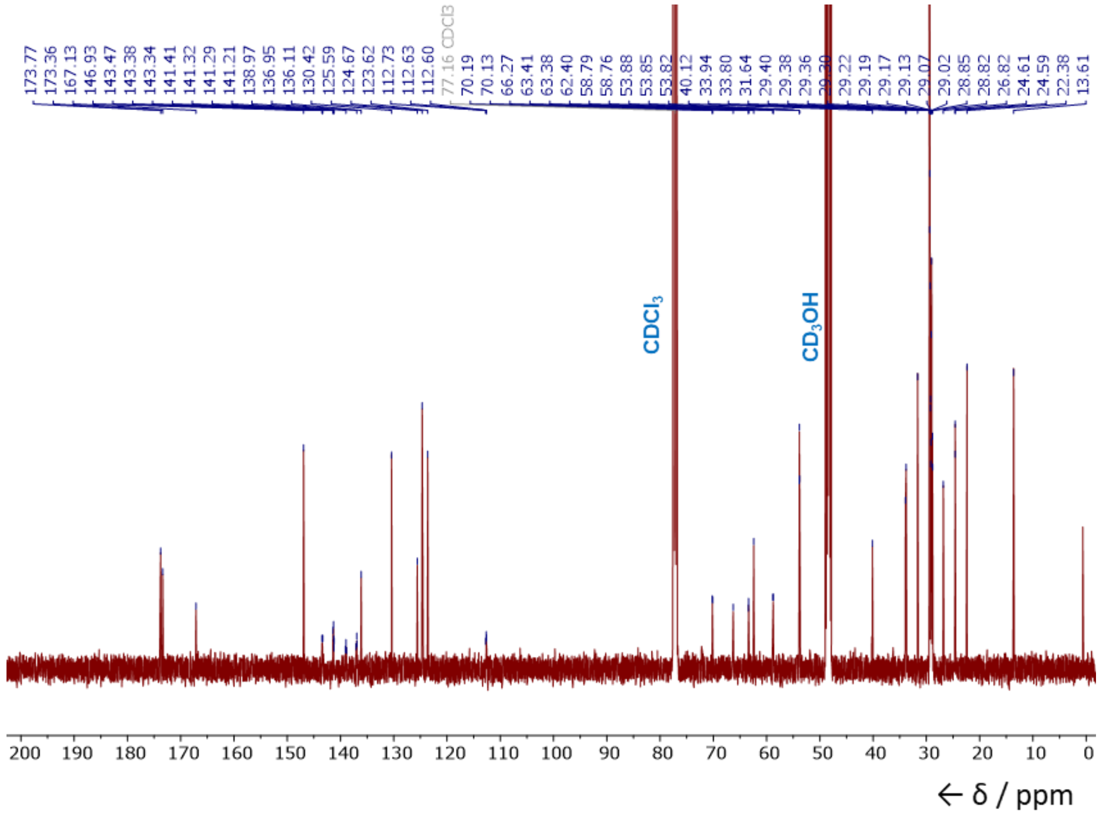

Figure S14. *^{13}C NMR spectrum of compound **1·HB** ($CDCl_3$:CD_3OD (2:1), 126 MHz, 298 K).*

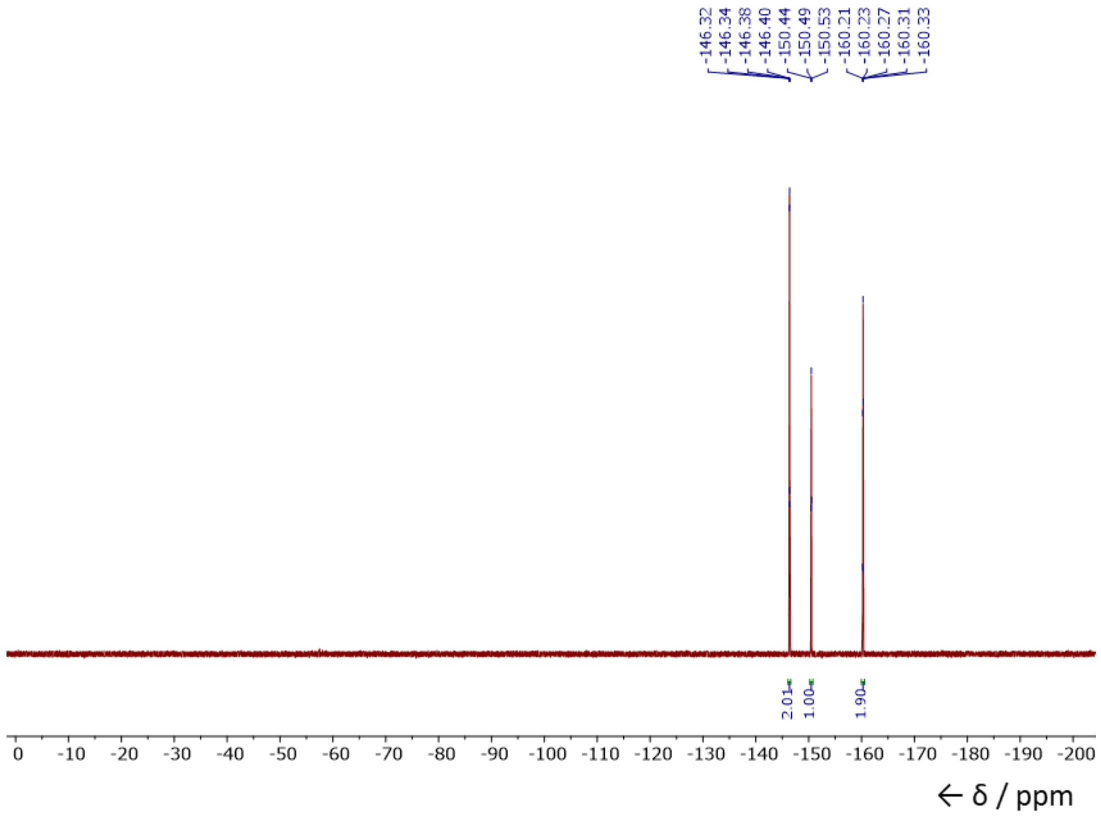

Figure S15. *^{19}F NMR spectrum of compound **1·HB** ($CDCl_3$:CD_3OD (2:1), 470 MHz, 298 K).*

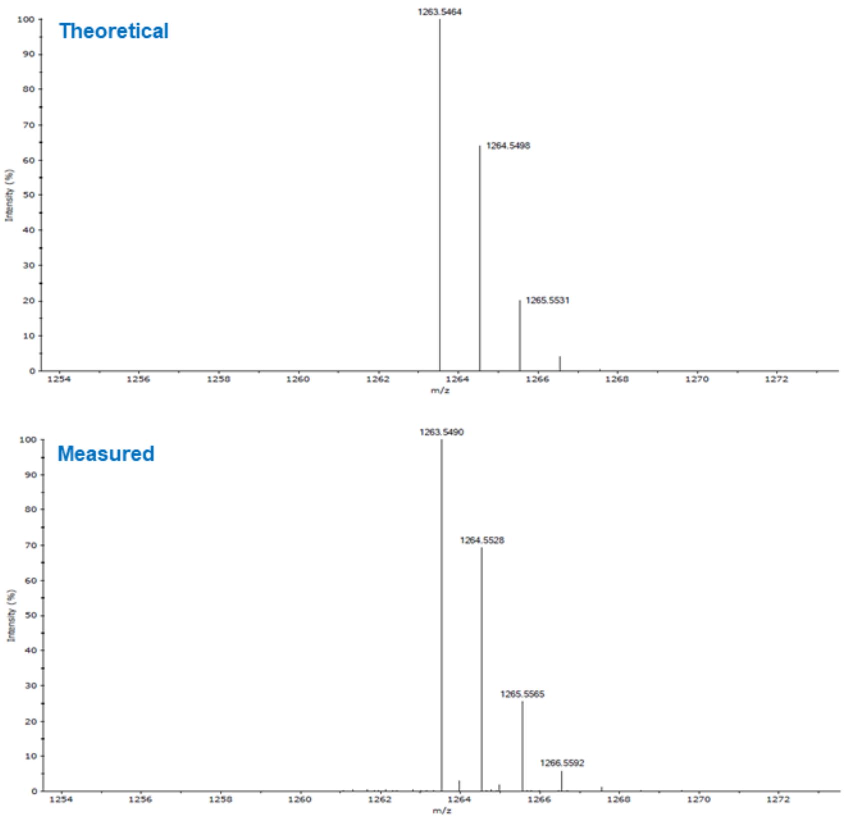

Figure S16. *HRMS spectrum of compound **1·HB**.*

2.6 Halogen bonding mobile carrier, 2·XB

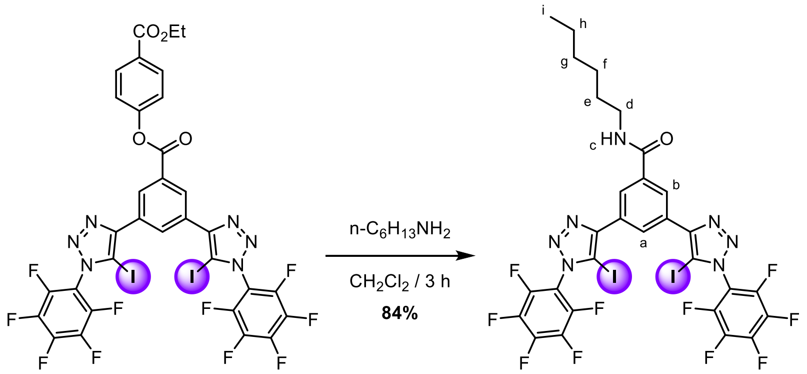

S2·XB **2·XB**

S2·XB (75 mg, 0.0759 mmol) and hexylamine (23 mg, 0.228 mmol) were dissolved in CH_2Cl_2 and left to stir at room temperature for 3 hours, after which time the mixture was diluted with CH_2Cl_2 (ca. 100 ml) and washed with $NH_4Cl_{(aq)}$ (50 ml) and purified by silica gel column chromatography (EtOAc:CH_2Cl_2, 2:98, v/v) and isolated as a white solid (59 mg, 0.0638 mmol, 84%).

^{1}H NMR (500 MHz, $CDCl_3$) δ 8.92 (t, J = 1.7 Hz, 1H, H_a), 8.59 (d, J = 1.7 Hz, 2H, H_b), 6.38 (t, J = 5.7 Hz, 1H, H_c), 3.53 (td, J = 7.3, 5.5 Hz, 2H, H_d), 1.82 – 1.09 (m, 8H, H_{e-h}), 0.96 – 0.83 (m, 3H, H_i).

^{19}F NMR (470 MHz, $CDCl_3$) δ -139.61 – -144.10 (m), -144.60 – -148.52 (m), -158.86 (dd, J = 20.9, 16.2 Hz).

^{13}C NMR (126 MHz, $CDCl_3$) δ 166.3, 149.5, 136.3, 130.5, 128.4, 126.5, 81.1, 40.6, 31.7, 29.8 (d, J = 7.0 Hz), 26.9, 22.7, 14.2 (Some signals coincident/non-observable due to C-F coupling.).

HRMS-ESI (m/z) Calculated for $C_{29}H_{18}F_{10}ON_7I_2$ $[M+H]^+$, 923.9497; found 923.9497.

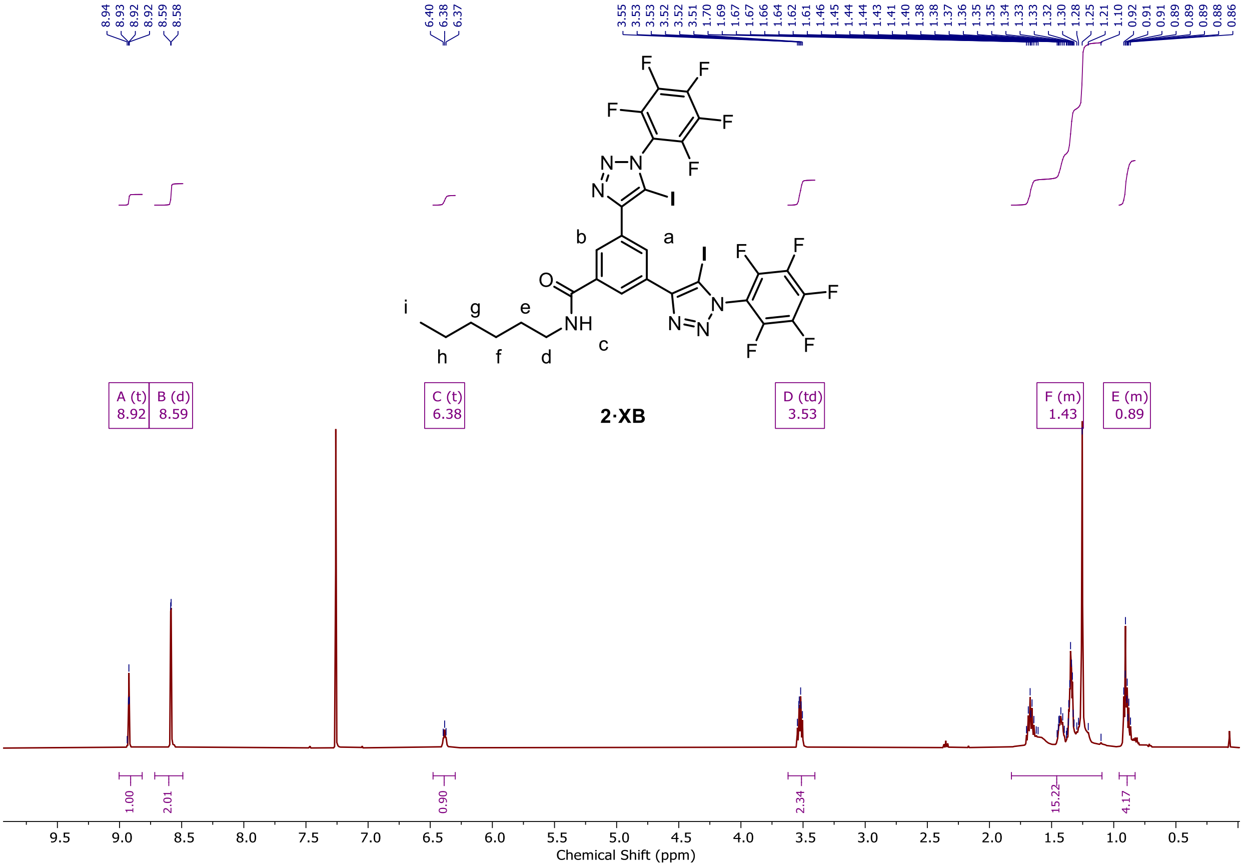

Figure S17. *^{1}H NMR spectrum of compound* ***2·XB*** *(CDCl₃, 400 MHz, 298 K).*

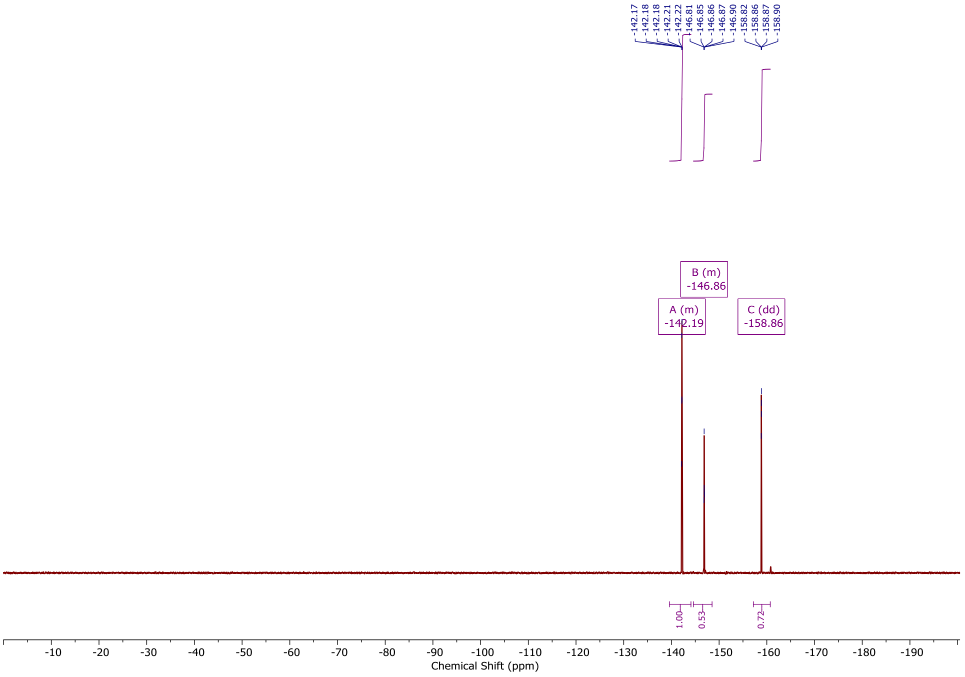

Figure S18. *^{19}F NMR spectrum of compound* ***2·XB*** *(CDCl₃, 470 MHz, 298 K).*

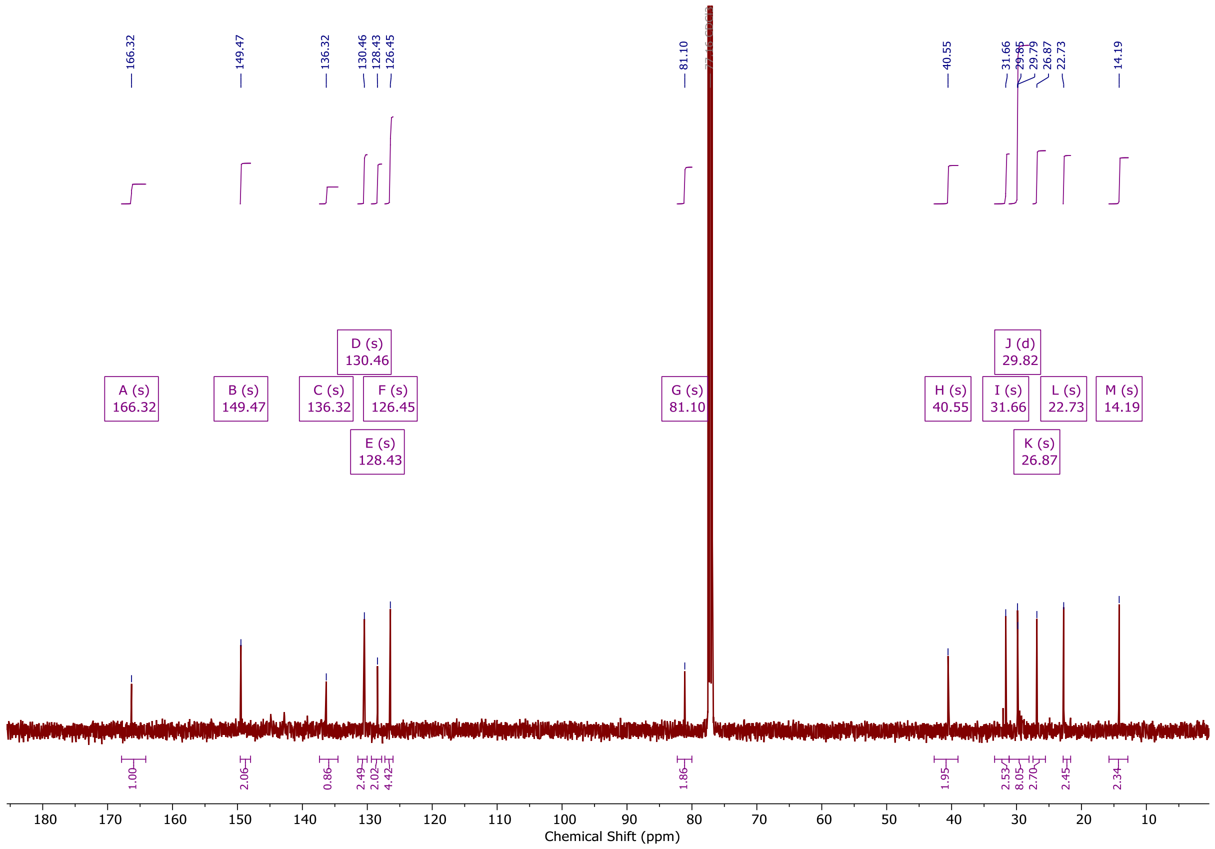

Figure S19. *^{13}CNMR spectrum of compound **2·XB** ($CDCl_3$, 126 MHz, 298 K).*

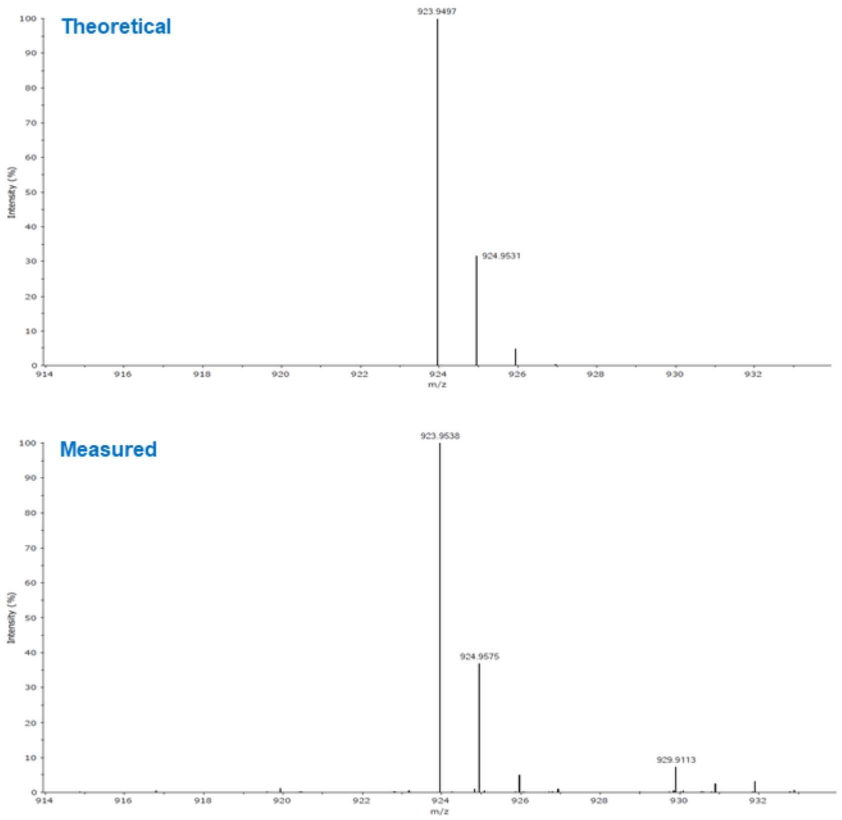

Figure S20. *HRMS spectrum of compound **2·XB**.*

3 UV-Visible Absorption Analysis

3.1 Determination of incorporation efficiency

UV-vis analysis was employed to determine the incorporation efficiency of the relay transporter **1·XB** into the membrane of pre-formed 200 nm POPC LUVs. First, the solution phase UV-vis of **1·XB** was measured in DMSO. The following procedure was then carried out to determine the incorporation efficiency of **1·XB**:

To 2.5 mL of POPC LUVs (200 nm, 0.50 mM lipid; internal and external buffer comprising 100 mM NaCl, 10 mM HEPES, pH 7.0) was added compound **1·XB** in DMSO (1.25 mM, 50 µL, 5 mol% to lipid) and stirred for 10 min. A 0.5 mL aliquot of this solution was diluted to a final volume of 1.0 mL with buffer solution (0.5 mL, 100 mM NaCl, 10 mM HEPES, pH 7.0) to give the 'pre-size exclusion sample.' Another 1.75 mL aliquot of the initial solution was eluted through a size exclusion Sephadex G-25 column with buffer solution (100 mM NaCl, 10 mM HEPES, pH 7.0) collecting the vesicles in 3.5 mL of solvent to give the 'post-size exclusion sample'; with the same total concentration of vesicles. The UV-vis spectra were recorded with no baseline correction and compared to the spectrum of POPC LUVs (200 nm, 0.25 mM lipid, 100 mM NaCl, 10 mM HEPES, pH 7.0).

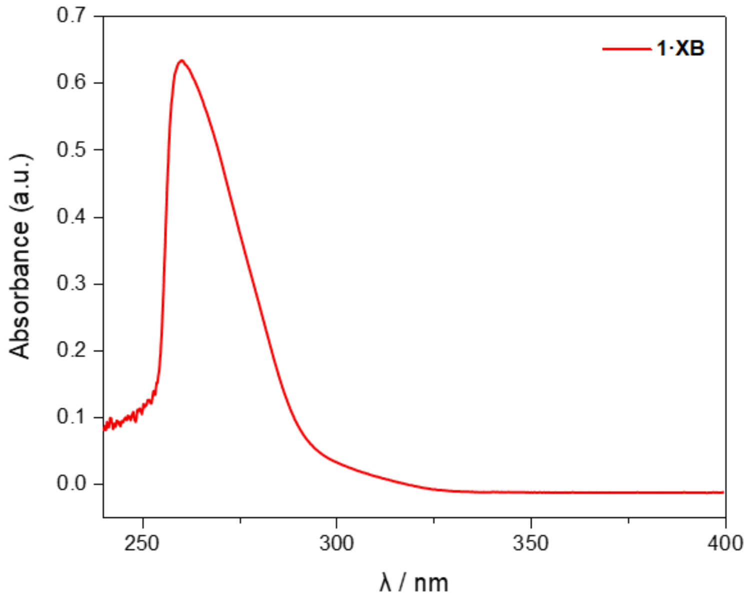

Figure S21. *UV-vis spectra of **1·XB** (15 µM) in DMSO.*

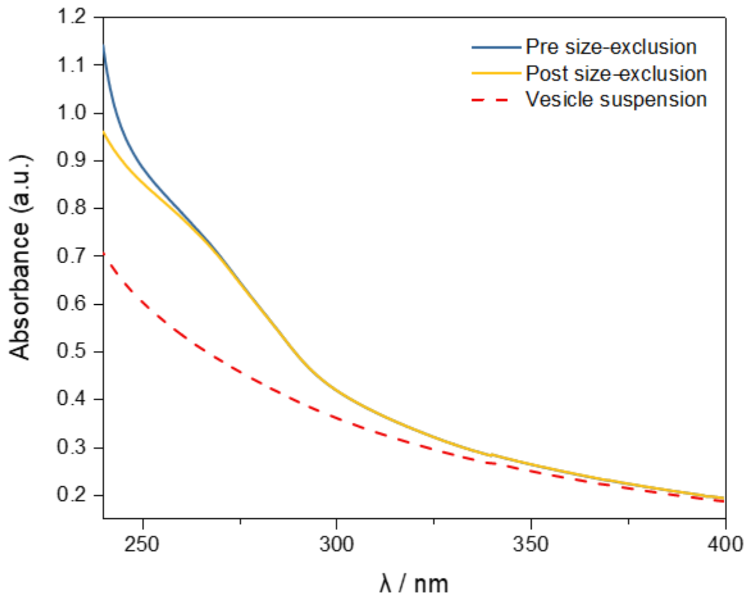

Figure S22. *UV-vis spectra of **1·XB** (5 mol% to lipid) embedded in POPC LUVs pre- and post-size exclusion. Incorporation efficiency was determined to be >95%.*

4 Anion Transport Experiments

4.1 Vesicle Preparation

A thin film of lipid (2.1875 μmol) and transporter in various ratios was formed by evaporating a chloroform solution under reduced pressure on a rotary evaporator (20 °C) and then under high vacuum for 6 hours. The lipid film was hydrated by vortexing with the prepared buffer (1 mL, 100 mM NaCl, 10 mM HEPES, 1 mM 8-hydroxypyrene-1,3,6-trisulfonic acid trisodium salt (HPTS), pH 7.0). The lipid suspension was then subjected to 5 freeze-thaw cycles using liquid nitrogen and a water bath (40°C) followed by extrusion 19 times through a polycarbonate membrane (pore size 200 nm). Extrusion was performed at 50°C in the case of DPPC lipids. Extra-vesicular components were removed by size exclusion chromatography on a Sephadex G-25 column eluted with 100 mM NaCl, 10 mM HEPES, pH 7.0. Final conditions: LUVs (0.625 mM lipid, 3.5 mL vesicle suspension); inside 100 mM NaCl, 10 mM HEPES, 1 mM HPTS, pH 7.0; outside: 100 mM NaCl, 10 mM HEPES, pH 7.0. Vesicles for the sodium gluconate assay were prepared by the same procedure, substituting NaCl for NaGluconate in the buffer solution.

4.2 Transport assays with HPTS

In a typical experiment, the LUVs containing HPTS with transporter pre-incorporated (100 μL, final lipid concentration 31.25 μM) were added to buffer (1880 μL of 100 mM NaCl, 10 mM HEPES, pH 7.0) at 25°C under gentle stirring. A pulse of NaOH (20 μL of 0.5 M solution, final concentration 5 mM) was added at 10 s to initiate the experiment. After 210 s, detergent (25 μL of 11% Triton X-100 in 7:1 (v/v) H_2O-DMSO) was added to lyse the vesicles and calibrate the assay. The fluorescence emission was monitored at λ_{em} = 510 nm (λ_{ex} = 405/460 nm). The fractional fluorescence intensity (I_{rel}) was calculated from Equation S1, where R_t is the fluorescence ratio at time t, (ratio of intensities from 460 nm / 405 nm excitation) R_0 is the fluorescence ratio at time 0 s immediately before the base pulse, and R_d is the fluorescence ratio at time 255 s, after the addition of detergent. For each compound, each individual concentration was repeated at least three times and averaged; error bars represent standard deviations.

$$I_{rel} = \frac{R_t - R_0}{R_d - R_0} \quad \textbf{(Equation S1)}$$

The fractional fluorescence intensity (I_{rel}) at 205 s just prior to lysis, defined as the fractional activity y, was plotted as a function of the ionophore concentration (x / μM). Hill coefficients (n) and EC_{50} values were calculated by fitting to the Hill equation (S2):

$$y = y_0 + (y_{max} - y_0)\frac{x^n}{EC_{50} + x^n} \quad \textbf{(Equation S2)}$$

The k_{ini} values were calculated by fitting the transport kinetics curves (I_{rel} vs t) with the exponential function I_{rel} = a – exp(-kt/b) using Origin 17. k_{ini} (the initial rate of transport at t = 10) is then given by differentiation as k_{ini} = 1/b (s^{-1}).

Experiments with DPPC lipids were conducted in the same way. For elevated temperature studies, the buffer was equilibrated at 45°C for 5 minutes prior to initiating the experiment.

Experiments in the presence of protonophore trifluoromethoxy carbonylcyanide phenylhydrazone (FCCP) were carried out using the above procedure, except that a DMSO solution of FCCP (5 μL of 100 μM solution, final concentration 0.25 μM/0.8 mol%) was added to the vesicle suspension prior to the addition of the NaOH pulse. At this concentration, FCCP does not cause appreciable dissipation of the transmembrane pH gradient alone.

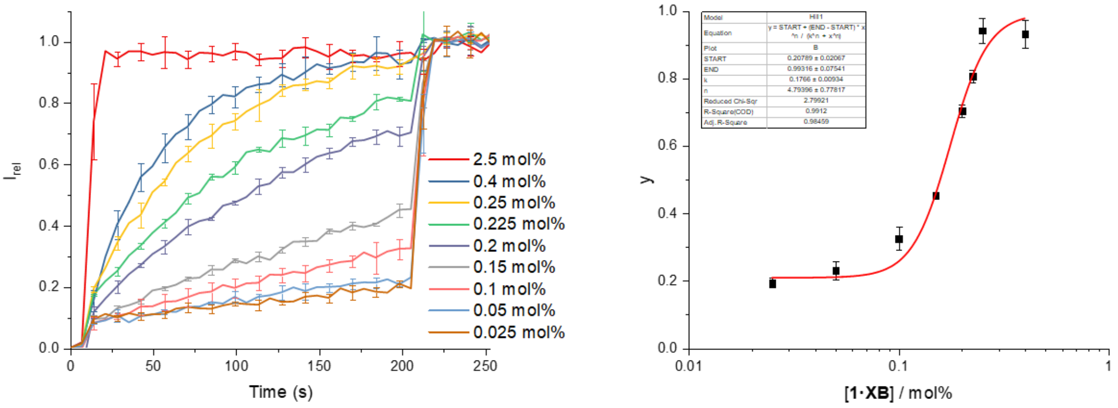

Figure S23. Left: *Ion transport HPTS assay data for* ***1·XB*** *pre-incorporated (0.025-2.5 mol% to lipid) in POPC LUVs.* ***Right:*** *Dependence of the fractional transport activity y in the HPTS assay on the concentration of* ***1·XB*** *(black squares) fitted to the Hill equation (red line).*

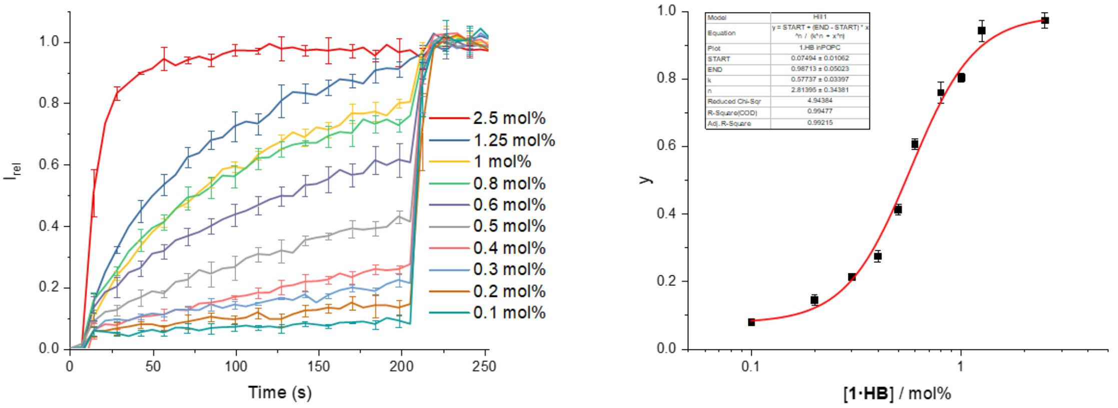

Figure S24. Left: *Ion transport HPTS assay data for* ***1·HB*** *pre-incorporated (0.1-2.5 mol% to lipid) in POPC LUVs.* ***Right:*** *Dependence of the fractional transport activity y in the HPTS assay on the concentration of* ***1·HB*** *(black squares) fitted to the Hill equation (red line).*

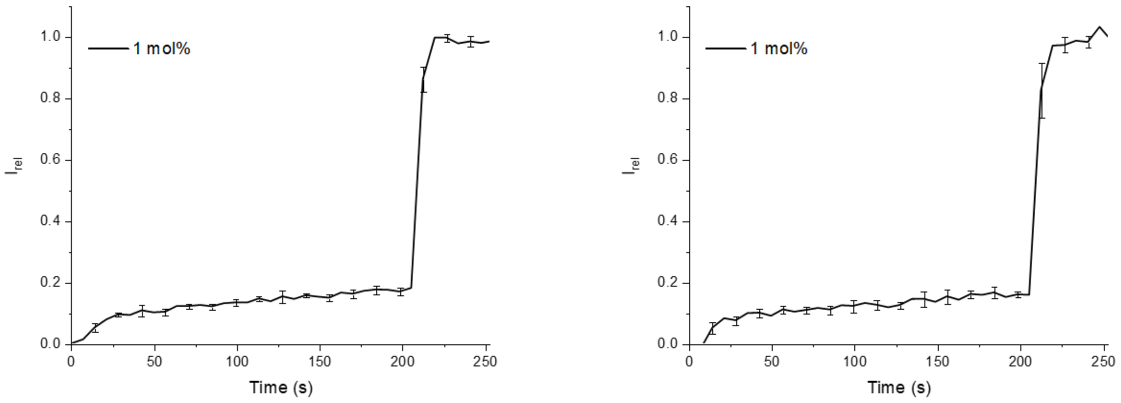

Figure S25. *Ion transport HPTS assay data for* ***1·XB*** *(left) and* ***1·HB*** *(right) externally added (5µL DMSO, 1 mol% to lipid) to POPC LUVs.*

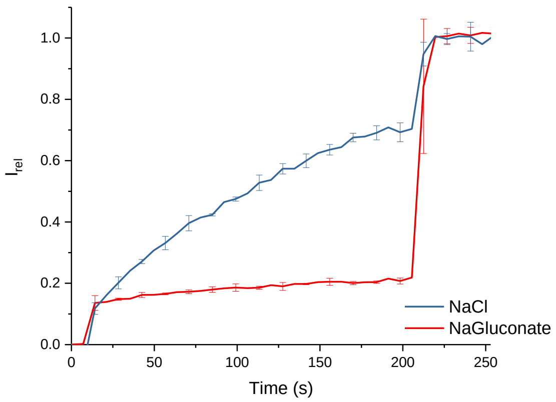

Figure S26. *Ion transport HPTS assay data for* ***1·XB*** *pre-incorporated (0.2 mol% to lipid) in POPC LUVs with NaGluconate buffer compared to NaCl buffer.*

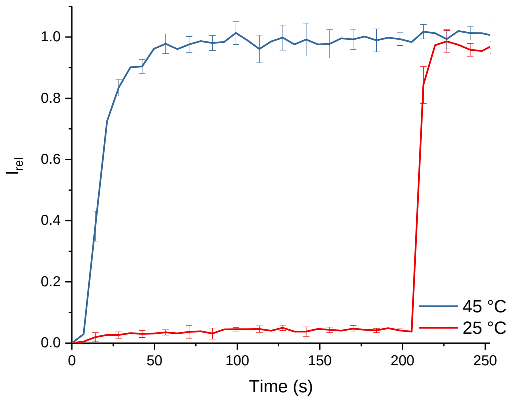

Figure S27. *Ion transport HPTS assay data for* ***1·XB*** *pre-incorporated (0.2 mol% to lipid) in DPPC LUVs at 25 °C and 45 °C.*

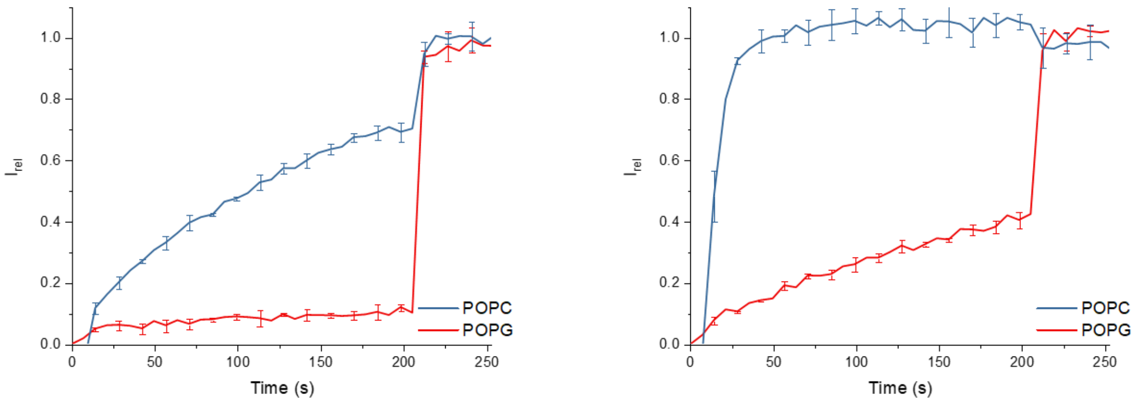

Figure S28. *Ion transport HPTS assay data for* ***1·XB*** *pre-incorporated (0.2 mol% to lipid) containing no FCCP (left) and with FCCP (right) (5 µL of 100 µM DMSO stock, 0.8 mol% to lipid) in POPG LUVs compared to POPC LUVs.*

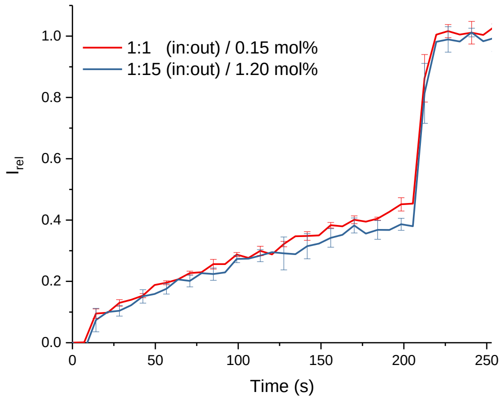

Figure S29. *Ion transport HPTS assay data for* ***1·XB*** *pre-incorporated with a symmetric distribution of relay transporters (red, 0.15 mol% to lipid pre-incorporated) and with an asymmetric distribution of relay transporters (blue, 0.15 mol% to lipid pre-incorporated with a further 1.05 mol% to total lipid added externally in 5µL DMSO).*

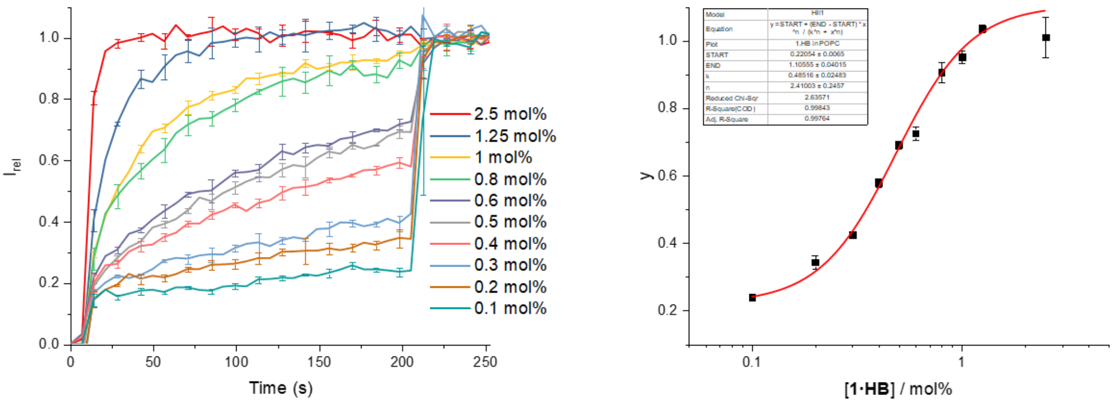

Figure S30. Left: *Ion transport HPTS assay data for **1·HB** pre-incorporated (0.1-2.5 mol% to lipid) in POPC LUVs with FCCP (5 µL of 100 µM DMSO stock, 0.8 mol% to lipid).* ***Right:*** *Dependence of the fractional transport activity y in the HPTS assay on the concentration of **1·HB** in the presence of FCCP (black squares) fitted to the Hill equation (red line).*

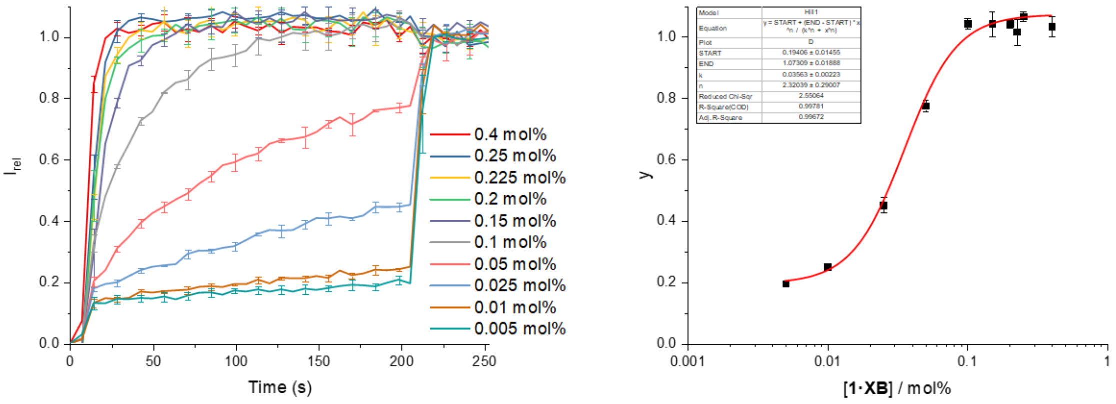

Figure S31. Left: *Ion transport HPTS assay data for **1·XB** pre-incorporated (0.005-0.4 mol% to lipid) in POPC LUVs with FCCP (5 µL of 100 µM DMSO stock, 0.8 mol% to lipid).* ***Right:*** *Dependence of the fractional transport activity y in the HPTS assay on the concentration of **1·XB** in the presence of FCCP (black squares) fitted to the Hill equation (red line).*

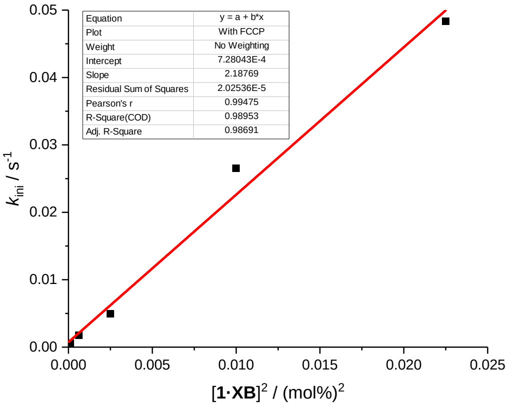

Figure S32. *Initial rates analysis of ion transport HPTS assay data for **1·XB** pre-incorporated (0.005-0.15 mol% to lipid) in POPC LUVs with FCCP (5 µL of 100 µM DMSO stock, 0.8 mol% to lipid). The initial rate, k_{ini}, is plotted against the concentration of **1·XB** squared, with the linear regression plotted.*

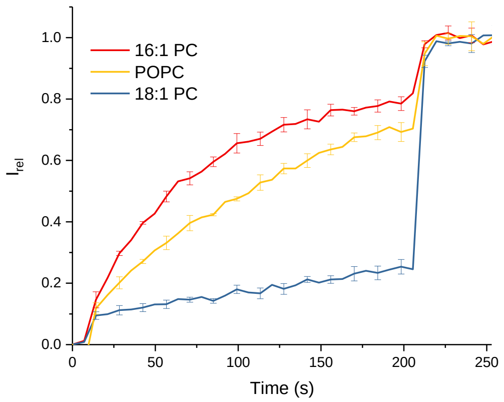

Figure S33. *Ion transport HPTS assay data for **1·XB** pre-incorporated (0.2 mol% to lipid) in different lipid LUVs (16:1 PC, POPC and 18:1 PC) to investigate the effect of changing the alkyl chain length.*

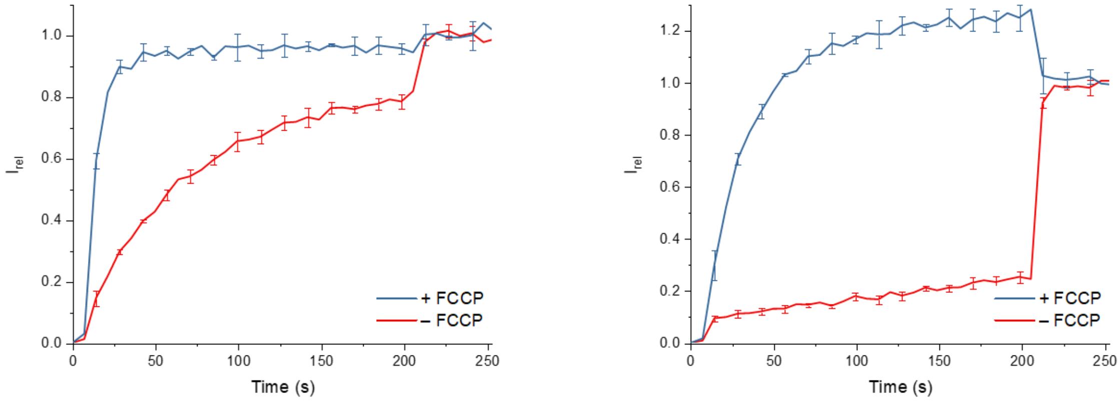

Figure S34. *Ion transport HPTS assay data for **1·XB** pre-incorporated (0.2 mol% to lipid) in 16:1 PC LUVs (left) and 18:1 PC LUVs (right) with FCCP (5 µL of 100 µM DMSO stock, 0.8 mol% to lipid) compared to no FCCP.*

For the mobile carrier transport experiments the procedure was slightly amended: A pulse of NaOH (20 μL of 0.5 M solution, final concentration 5 mM) was added at 40 s to initiate the experiment. At 90 s the test mobile carrier (various concentrations, in 5 μL DMSO) was added, followed by detergent (25 μL of 11% Triton X-100 in 7:1 (v/v) H_2O-DMSO) at 290 s to lyse the vesicles and calibrate the assay. The fluorescence emission was monitored at λ_{em} = 510 nm (λ_{ex} = 405/460 nm). The fractional fluorescence intensity (I_{rel}) was calculated from Equation S1, where R_t is the fluorescence ratio at time t, (ratio of intensities from 460 nm / 405 nm excitation) R_0 is the fluorescence ratio at time 65 s immediately before the base pulse, and R_d is the fluorescence ratio at time 350 s, after the addition of detergent. For each compound, each individual concentration was repeated at least twice and averaged; error bars represent standard deviations. Experiments in the presence of FCCP were carried out using this procedure, except that a DMSO solution of FCCP (5 μL of 100 μM solution, final concentration 0.25 μM/0.8 mol%) was added to the vesicle suspension prior to the start of the run. At this concentration, FCCP does not cause appreciable dissipation of the transmembrane pH gradient alone.

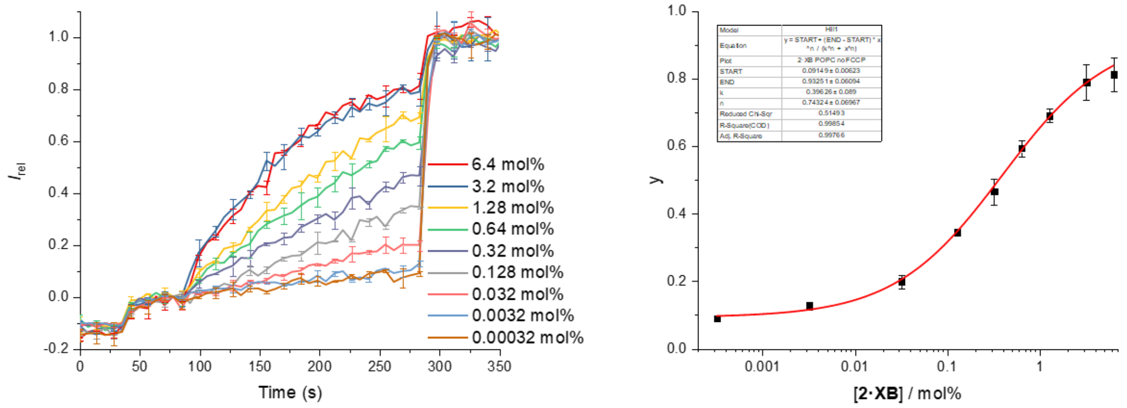

Figure S35. Left: *Ion transport HPTS assay data for **2·XB** externally added (5μL DMSO, 0.00032-6.4 mol% to lipid) to POPC LUVs.* ***Right:*** *Dependence of the fractional transport activity y in the HPTS assay on the concentration of **2·XB** (black squares) fitted to the Hill equation (red line).*

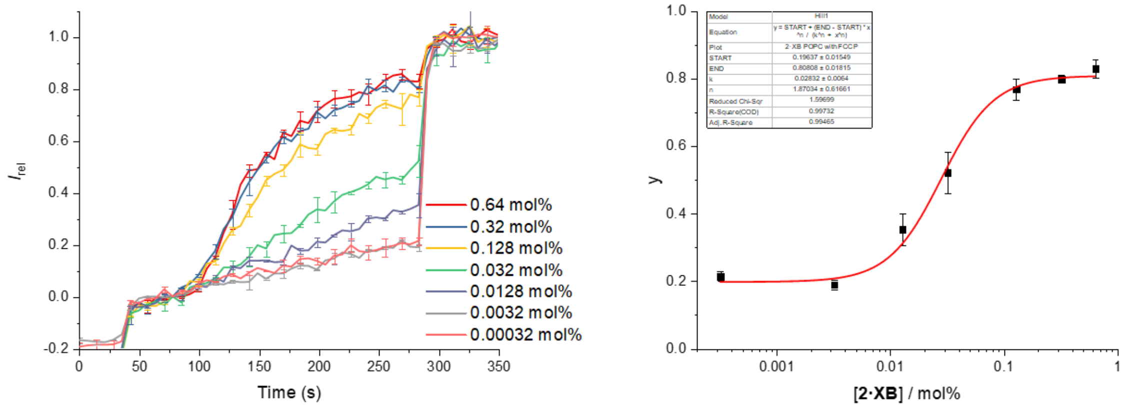

Figure S36. Left: *Ion transport HPTS assay data for **2·XB** externally added (5μL DMSO, 0.00032-0.64 mol% to lipid) to POPC LUVs with FCCP (5 μL of 100 μM DMSO stock, 0.8 mol% to lipid).* ***Right:*** *Dependence of the fractional transport activity y in the HPTS assay on the concentration of **2·XB** in the presence of FCCP (black squares) fitted to the Hill equation (red line).*

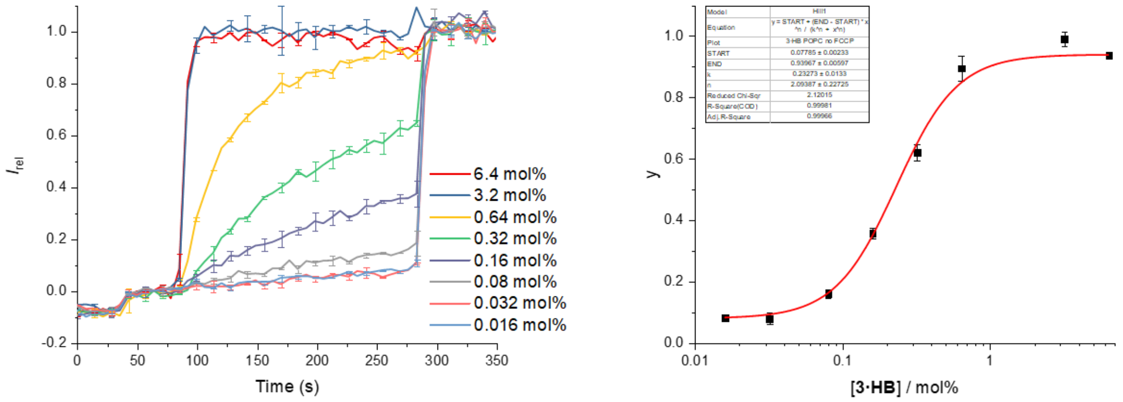

Figure S37. Left: *Ion transport HPTS assay data for* ***3·HB*** *externally added (5µL DMSO, 0.016-6.4 mol% to lipid) to POPC LUVs.* ***Right:*** *Dependence of the fractional transport activity y in the HPTS assay on the concentration of* ***3·HB*** *(black squares) fitted to the Hill equation (red line).*

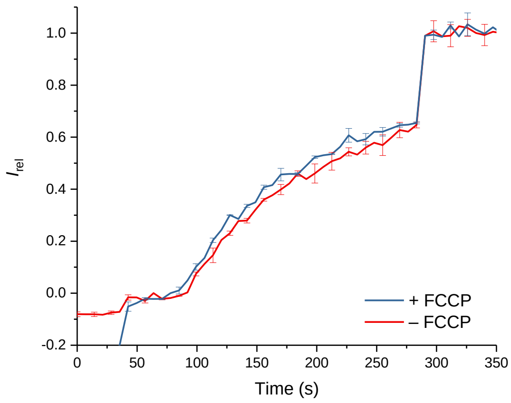

Figure S38. *Ion transport HPTS assay data for* ***3·HB*** *externally added (5µL DMSO, 0.32 mol% to lipid) to POPC LUVs with FCCP (5 µL of 100 µM DMSO stock, 0.8 mol% to lipid) compared to no FCCP.*

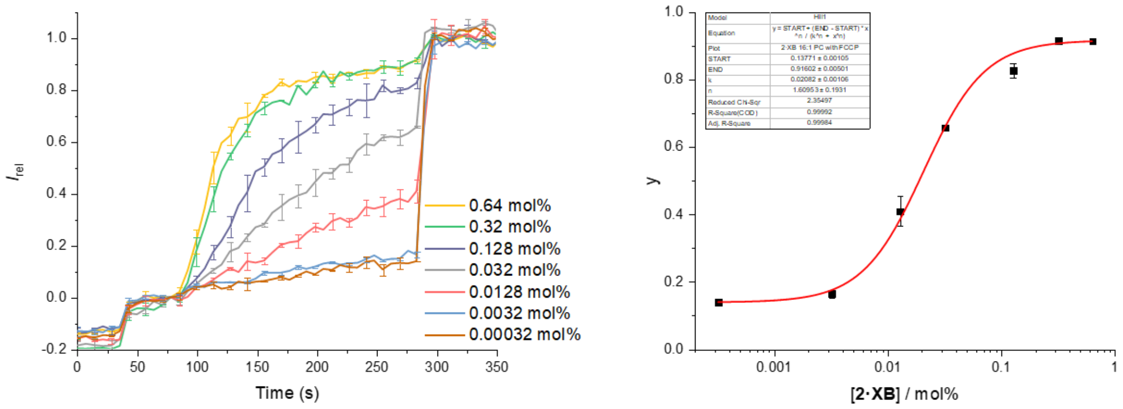

***Figure S39. Left:** Ion transport HPTS assay data for **2·XB** externally added (5µL DMSO, 0.00032-0.64 mol% to lipid) to 16:1 PC LUVs with FCCP (5 µL of 100 µM DMSO stock, 0.8 mol% to lipid). **Right:** Dependence of the fractional transport activity y in the HPTS assay on the concentration of **2·XB** in the presence of FCCP (black squares) fitted to the Hill equation (red line).*

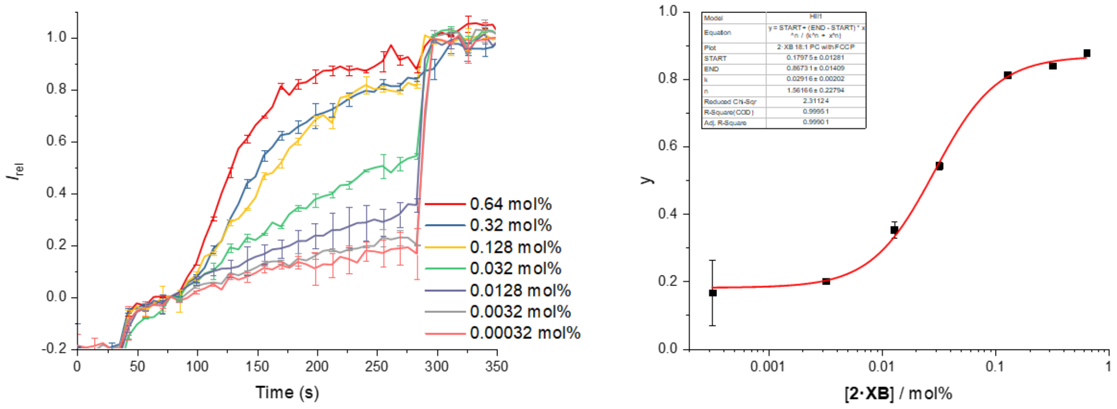

***Figure S40. Left:** Ion transport HPTS assay data for **2·XB** externally added (5µL DMSO, 0.00032-0.64 mol% to lipid) to 18:1 PC LUVs with FCCP (5 µL of 100 µM DMSO stock, 0.8 mol% to lipid). **Right:** Dependence of the fractional transport activity y in the HPTS assay on the concentration of **2·XB** in the presence of FCCP (black squares) fitted to the Hill equation (red line).*

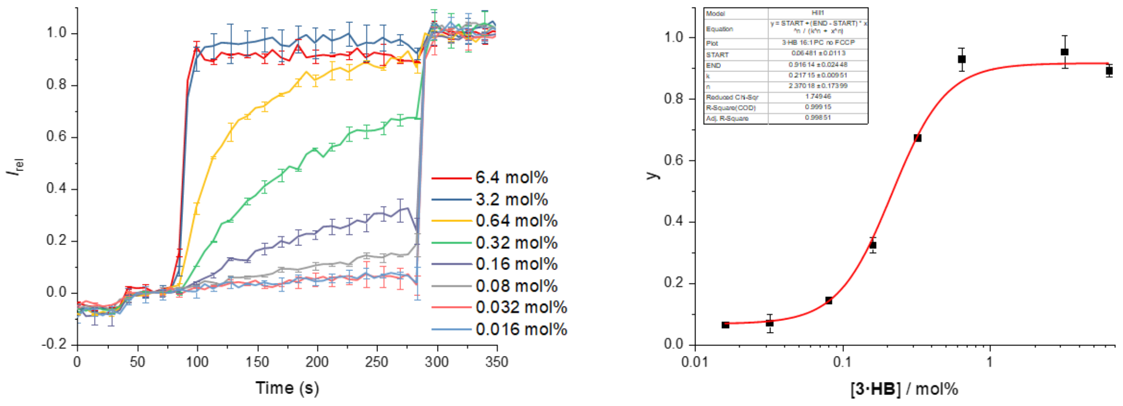

Figure S41. Left: *Ion transport HPTS assay data for* ***3·HB*** *externally added (5µL DMSO, 0.016-6.4 mol% to lipid) to 16:1 PC LUVs.* ***Right:*** *Dependence of the fractional transport activity y in the HPTS assay on the concentration of* ***3·HB*** *(black squares) fitted to the Hill equation (red line).*

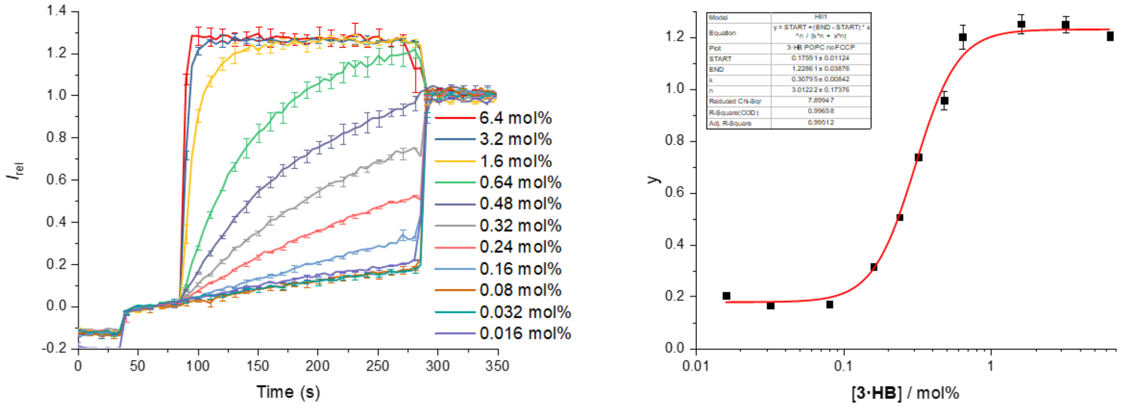

Figure S42. Left: *Ion transport HPTS assay data for* ***3·HB*** *externally added (5µL DMSO, 0.016-6.4 mol% to lipid) to 18:1 PC LUVs.* ***Right:*** *Dependence of the fractional transport activity y in the HPTS assay on the concentration of* ***3·HB*** *(black squares) fitted to the Hill equation (red line).*

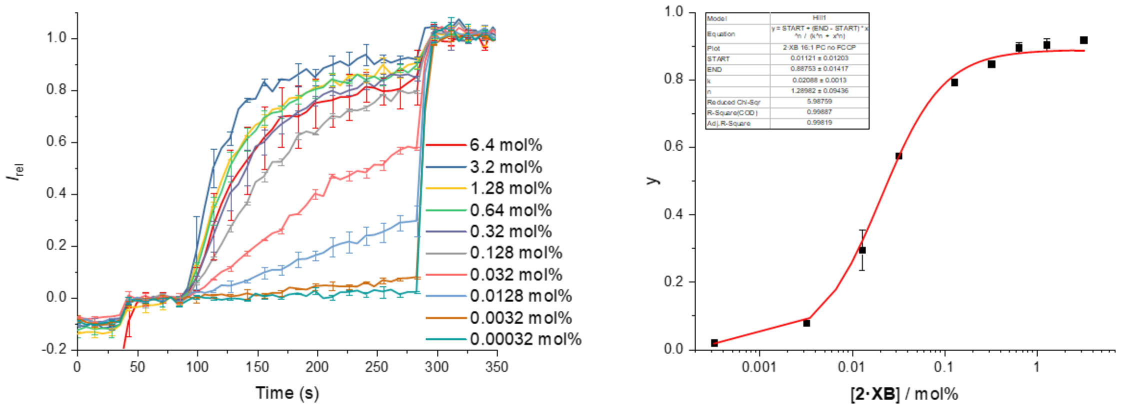

Figure S43. Left: *Ion transport HPTS assay data for **2·XB** externally added (5µL DMSO, 0.00032-6.4 mol% to lipid) to 16:1 PC LUVs.* ***Right:*** *Dependence of the fractional transport activity y in the HPTS assay on the concentration of **2·XB** (black squares) fitted to the Hill equation (red line).*

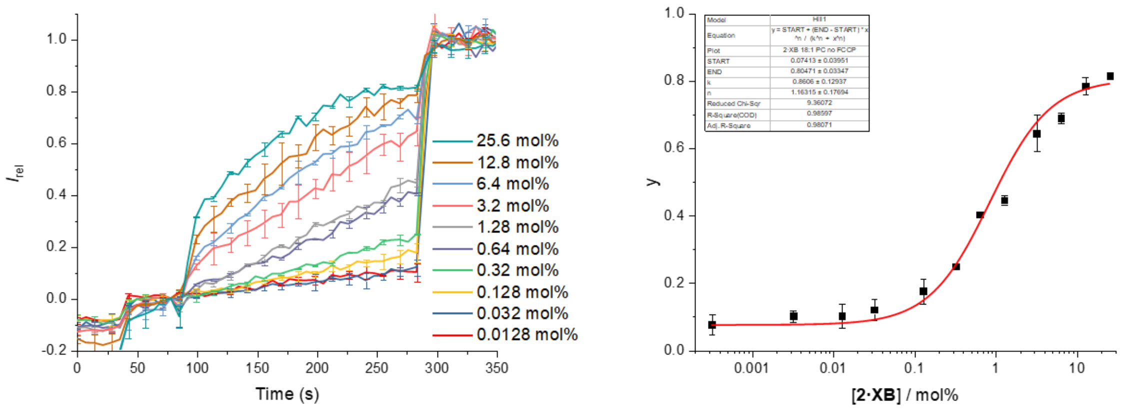

Figure S44. Left: *Ion transport HPTS assay data for **2·XB** externally added (5µL DMSO, 0.0128-25.6 mol% to lipid) to 18:1 PC LUVs.* ***Right:*** *Dependence of the fractional transport activity y in the HPTS assay on the concentration of **2·XB** (black squares) fitted to the Hill equation (red line).*

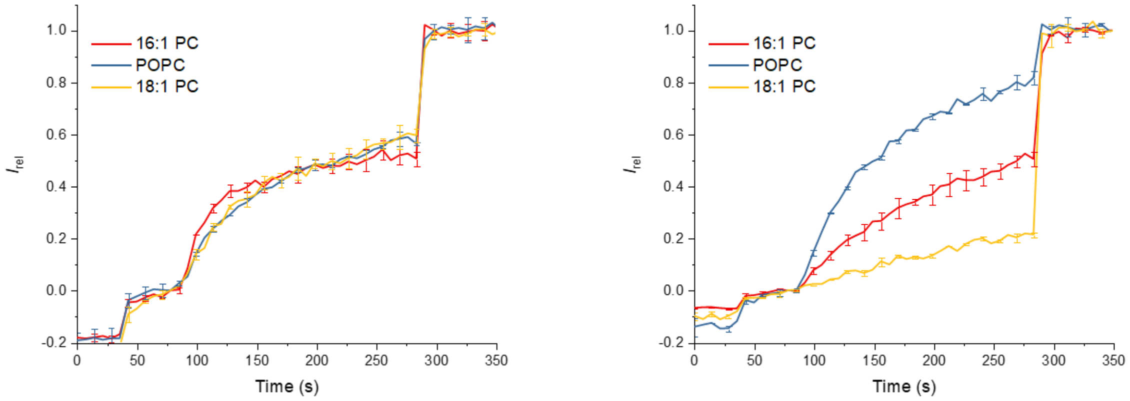

Figure S45. Left: *Ion transport HPTS assay data for **4·HB** externally added (5µL DMSO, 0.0032 mol% to lipid) to 16:1 PC, POPC and 18:1 PC LUVs with FCCP (5 µL of 100 µM DMSO stock, 0.8 mol% to lipid).* ***Right:*** *Ion transport HPTS assay data for **4·HB** externally added (5µL DMSO, 0.016 mol% to lipid) to 16:1 PC, POPC and 18:1 PC LUVs.*

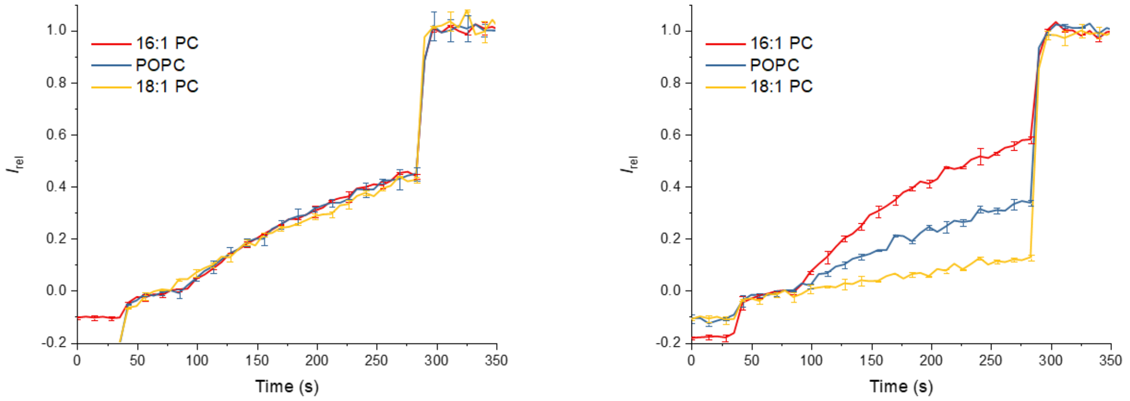

Figure S46. Left: *Ion transport HPTS assay data for **5·XB** externally added (5µL DMSO, 0.0032 mol% to lipid) to 16:1 PC, POPC and 18:1 PC LUVs with FCCP (5 µL of 100 µM DMSO stock, 0.8 mol% to lipid).* ***Right:*** *Ion transport HPTS assay data for **5·XB** externally added (5µL DMSO, 0.016 mol% to lipid) to 16:1 PC, POPC and 18:1 PC LUVs.*

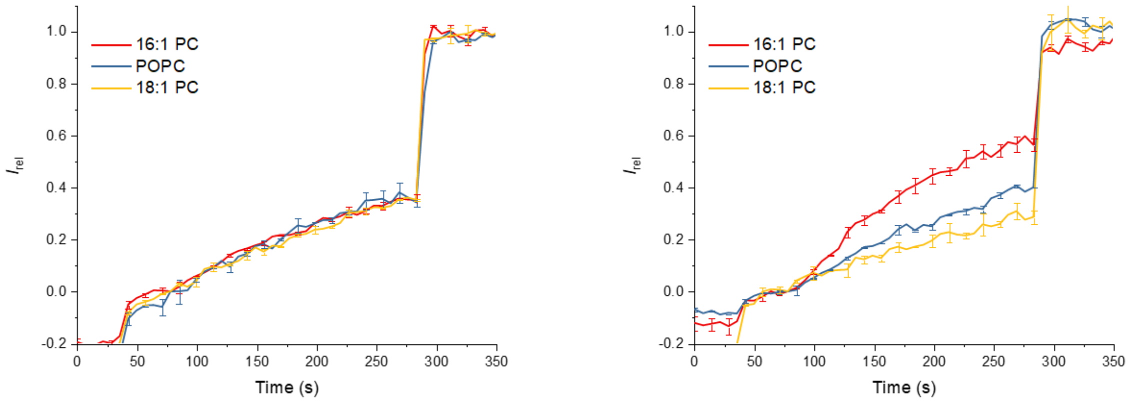

Figure S47. Left: *Ion transport HPTS assay data for* ***5·ChB*** *externally added (5µL DMSO, 0.01 mol% to lipid) to 16:1 PC, POPC and 18:1 PC LUVs with FCCP (5 µL of 100 µM DMSO stock, 0.8 mol% to lipid).* ***Right:*** *Ion transport HPTS assay data for* ***5·ChB*** *externally added (5µL DMSO, 0.1 mol% to lipid) to 16:1 PC, POPC and 18:1 PC LUVs.*

5 ^{1}H NMR Titration Experiments

Aliquots of anion ([TBACl] = 50 mM in acetone-d$_6$:D_2O, (v/v 97.5/2.5)) were added to an acetone-d$_6$ solution of the receptor ([Host] = 1 mM). Spectra were recorded at 0, 0.2, 0.4, 0.6, 0.8, 1.0, 1.2, 1.4, 1.6, 1.8, 2.0, 2.5, 3.0, 4.0, 5.0, 7.0 and 10 equivalents. Chemical shifts were referenced to the residual solvent peak.

K_a(Cl^-): 4660 (72) M^{-1}

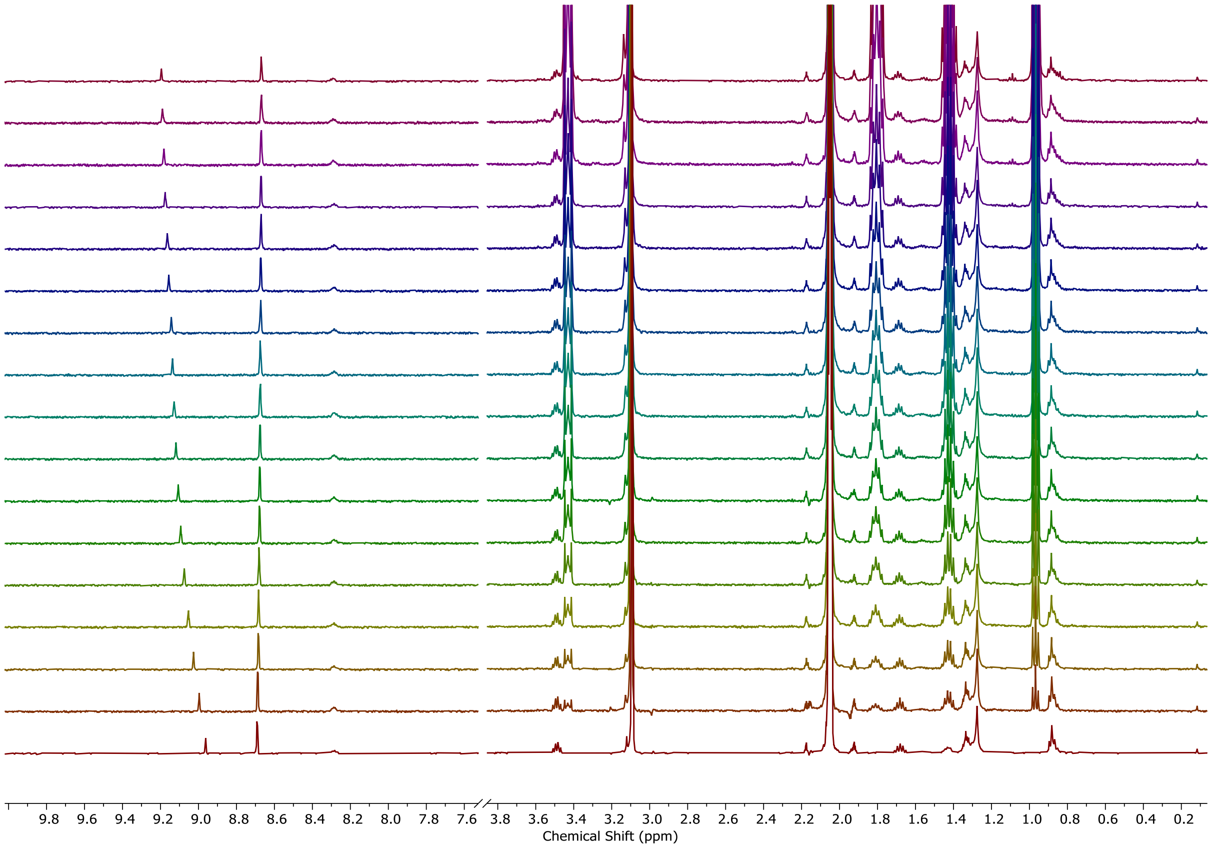

Figure S48. *Stacked* ^{1}*H NMR Anion binding titration isotherm for* ***2·XB*** *with TBACl.*

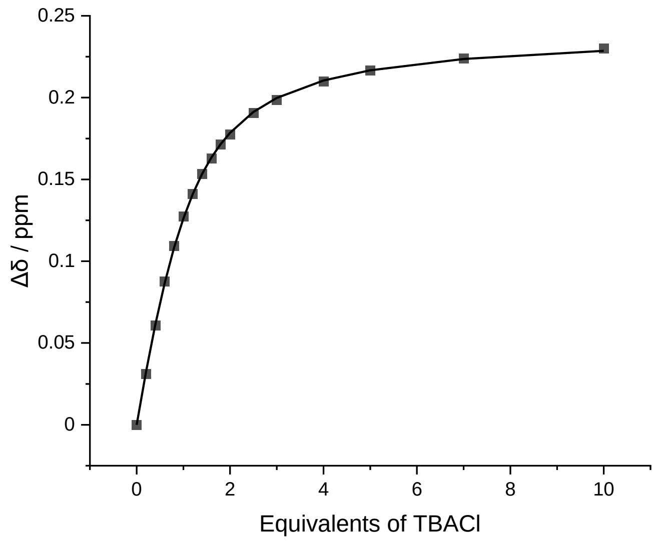

Figure S49. *Anion binding titration isotherm for **2·XB** with TBACl, solid points represent experimental data and the line represented fitted data.*

6 References

1 T. G. Johnson, A. Sadeghi-Kelishadi and M. J. Langton, *J. Am. Chem. Soc.*, 2022, **144**, 10455–10461.

2 A. Docker, J. G. Stevens and P. D. Beer, *Chem. - A Eur. J.*, 2021, **27**, 14600–14604.

3 S. J. Moore, M. Wenzel, M. E. Light, R. Morley, S. J. Bradberry, P. Gómez-Iglesias, V. Soto-Cerrato, R. Pérez-Tomás and P. A. Gale, *Chem. Sci.*, 2012, **3**, 2501–2509.

4 X. Wu, L. W. Judd, E. N. W. Howe, A. M. Withecombe, V. Soto-Cerrato, H. Li, N. Busschaert, H. Valkenier, R. Pérez-Tomás, D. N. Sheppard, Y. B. Jiang, A. P. Davis and P. A. Gale, *Chem*, 2016, **1**, 127–146.

5 L. E. Bickerton, A. Docker, A. J. Sterling, H. Kuhn, F. Duarte, P. D. Beer and M. J. Langton, *Chem. - A Eur. J.*, 2021, **27**, 11738–11745.
